# Human factors validation study of an artificial neural network‑based preoperative decision‑support tool for noninvasive lymph node staging (NILS) in women with primary breast cancer (ISRCTN99301435)

**DOI:** 10.1186/s12885-026-16161-5

**Published:** 2026-05-28

**Authors:** Anna Allfelt, Pär-Ola Bendahl, Looket Dihge, Mattias Ohlsson, Lisa Rydén, Ida Skarping

**Affiliations:** 1 Department of Surgery and Gastroenterology, Skåne University Hospital, Malmö, Sweden; 2 Department of Clinical Sciences, Division of Surgery, Lund University, Lund, Sweden; 3 Department of Clinical Sciences, Division of Oncology, Lund University, Lund, Sweden; 4 Department of Plastic and Reconstructive Surgery, Skåne University Hospital, Malmö, Sweden; 5 Department of Earth and Environmental Sciences, Lund University, Lund, Sweden; 6 Centre for Applied Intelligent Systems Research, Halmstad University, Halmstad, Sweden; 7 Department of Clinical Physiology and Nuclear Medicine, Skåne University Hospital, Lund, Sweden

**Keywords:** Breast neoplasm, Staging, Axillary lymph nodes, Decision aid, Sentinel lymph node biopsy, Human factors validation study

## Abstract

**Background:**

The integration of clinical decision support tools in medical practice is challenging and must be carefully undertaken, especially in cancer management. Noninvasive Lymph Node Status (NILS) is a web-based tool designed to estimate the probability of benign axillary lymph nodes in female patients with breast cancer scheduled for primary surgery. The aim was to identify barriers to NILS adoption in a clinical setting and assess whether intended users can operate the tool without significant errors or difficulties. Additionally, the study aimed to evaluate the appropriateness of result interpretation for decisions to abstain from sentinel lymph node biopsy (SNLB) and measure overall user satisfaction with the tool.

**Methods:**

This mixed-methods multicenter on-site qualitative human factor validation study was conducted in a simulated clinical environment, replicating both real-world physical and digital conditions. Based on the identified target user population for the NILS model, twenty physicians comprised the cohort. The study used simulated clinical cases, the System Usability Scale (SUS), and the After-Scenario Questionnaire (ASQ) to evaluate usability and satisfaction. An oral interview was conducted after the evaluations. The study followed a structured protocol, with distinct roles assigned to the test participants, leader, and observer.

**Results:**

Twenty physicians from four hospitals, with a median of 9.5 years of specialist practice, participated. Most participants were surgeons (75%, *N* = 15), while the remaining 25% (*N* = 5) were oncologists. Usability scores were high, with a mean SUS score of 89.5 (“excellent”) and a mean ASQ score of 6.3 (Likert scale 1–7). Several interface challenges were identified, including difficulty in locating the reset and information buttons, the ability to enter values outside valid ranges (age and tumor size), and the risk of unintentionally modifying the entered values while scrolling.

**Conclusions:**

This study identified the key usability factors, barriers, and facilitators affecting the NILS model implementation. Physicians found the NILS interface easy to use and valued the presented risk estimates of benign axillary lymph node status in their decisions to perform or abstain from SLNB. Study-informed interface redesigns have been implemented. Iterative usability work in clinical settings is crucial for successful implementation.

**Trial registration:**

ISRCTN99301435.

**Supplementary Information:**

The online version contains supplementary material available at 10.1186/s12885-026-16161-5.

## Introduction

The introduction of decision support tools in clinical medicine is challenging and must be carefully undertaken, especially in critical situations such as cancer management. The consideration of human factors, such as usability, in a clinical setting is crucial for successful implementation.

Breast cancer is the most common cancer affecting women, with 2.3 million new cases worldwide annually [1]. The majority of patients are diagnosed with early-stage breast cancer, and only 20–30% present with nodal metastases in the axilla—a rate that has been declining during the last decades [2, 3]. As a tool to support adjuvant treatment recommendations, the sentinel lymph node biopsy (SLNB) procedure is the gold standard for nodal staging in patients with clinically node-negative breast cancer (cN0) [4]. However, recent de-escalation approaches have questioned the necessity of SLNB for all patients, as suggested by the American Society of Clinical Oncology (ASCO) guidelines presented in 2021 [5]. Supporting this notion, the randomized non-inferiority SOUND trial demonstrated equal 5-year distant disease-free survival in patients with early cN0 breast cancer with small tumors (≤ 2 cm) randomized to either SLNB (0.4% axillary recurrences as a first event) or its omission (0.7% axillary recurrences a first event) [6]. The randomized non-inferiority INSEMA trial demonstrated that omitting SLNB is noninferior to performing SLNB, even in cases involving larger tumors (< 5 cm). The trial showed equivalent 6-year invasive disease-free survival rates in the study arms, although the incidence of axillary recurrence as a first clinical event was 1.0% in the SLNB-omission group and 0.3% in the SLNB group [7]. Additionally, the ASCO guidelines support a case-by-case evaluation of omitting SLNB, as initially proposed by the authors of the Choosing Wisely guidelines [5]. 

The Noninvasive Lymph Node Status (NILS) model is a web-based tool designed to estimate the probability of benign axillary lymph nodes in women with primary T1-2 invasive breast cancer with cN0 scheduled for primary surgery [8]. By using preoperative patient data and tumor characteristics, the machine learning-based NILS-algorithm provides a noninvasive prediction distinguishing node negative (N0) from node positive (N+) with a cut-off acknowledging a false-negative rate of 10% which is clinically accepted for the standard SLNB-technique [9, 10]. Model development showed that tumor size was the most important nodal status predictor, followed by nine clinicopathological variables including age, whereas HER2 status was excluded in the model due to limited predictive contribution and histological grade was omitted due to strong correlation to Ki67 [11]. The NILS model, therefore, provides support to attending physicians in deciding whether to perform or abstain from SLNB during primary breast cancer surgery. Introduced in 2019, the NILS model has been validated across geographical and temporal cohorts, in addition to when utilizing preoperatively-available data only [12, 13]. A health-economic decision-analytic model demonstrated that implementing the NILS model could lead to significant cost reductions and potential overall health benefits, particularly for patients undergoing breast-conserving surgery [14]. The NILS model is not yet CE-marked and is not available on the market.

This study aimed to identify barriers to the NILS model use in a simulated clinical setting, assess whether intended users could operate the NILS model without significant errors or difficulties, evaluate the appropriateness of result interpretation for decisions to abstain from SLNB, and measure overall user satisfaction with the tool.

## Methods

The NILS calculator was evaluated in this premarket mixed-methods human factor validation test under simulated conditions. Only the calculator interface was included in the scope of this study, while access and information pages were not.

Before data collection, the study was registered in the ISRCTN registry (ISRCTN99301435, registration date 15th Nov, 2024). The Consolidated Criteria for Reporting Qualitative Research checklist was used in this qualitative study [15]. 

### The NILS model - user interface

The technical details of the NILS algorithm and the validation processes have been published elsewhere [8, 12, 13]. Herein, the usability aspects are described.

#### Graphical representation of device and user interface

The device is a webpage (https://nils.cec.lu.se/) that is accessible via a browser on a computer, tablet, or smartphone. Upon logging in, users are greeted by an information page regarding the NILS model, which includes disclaimer information. The calculator can be accessed by clicking the “To calculator” button or by selecting “Calculator” from the menu. The menu also includes options for additional information about the NILS model, details about the research and the team, disclaimers, and contact details.

**Fig. 1:**
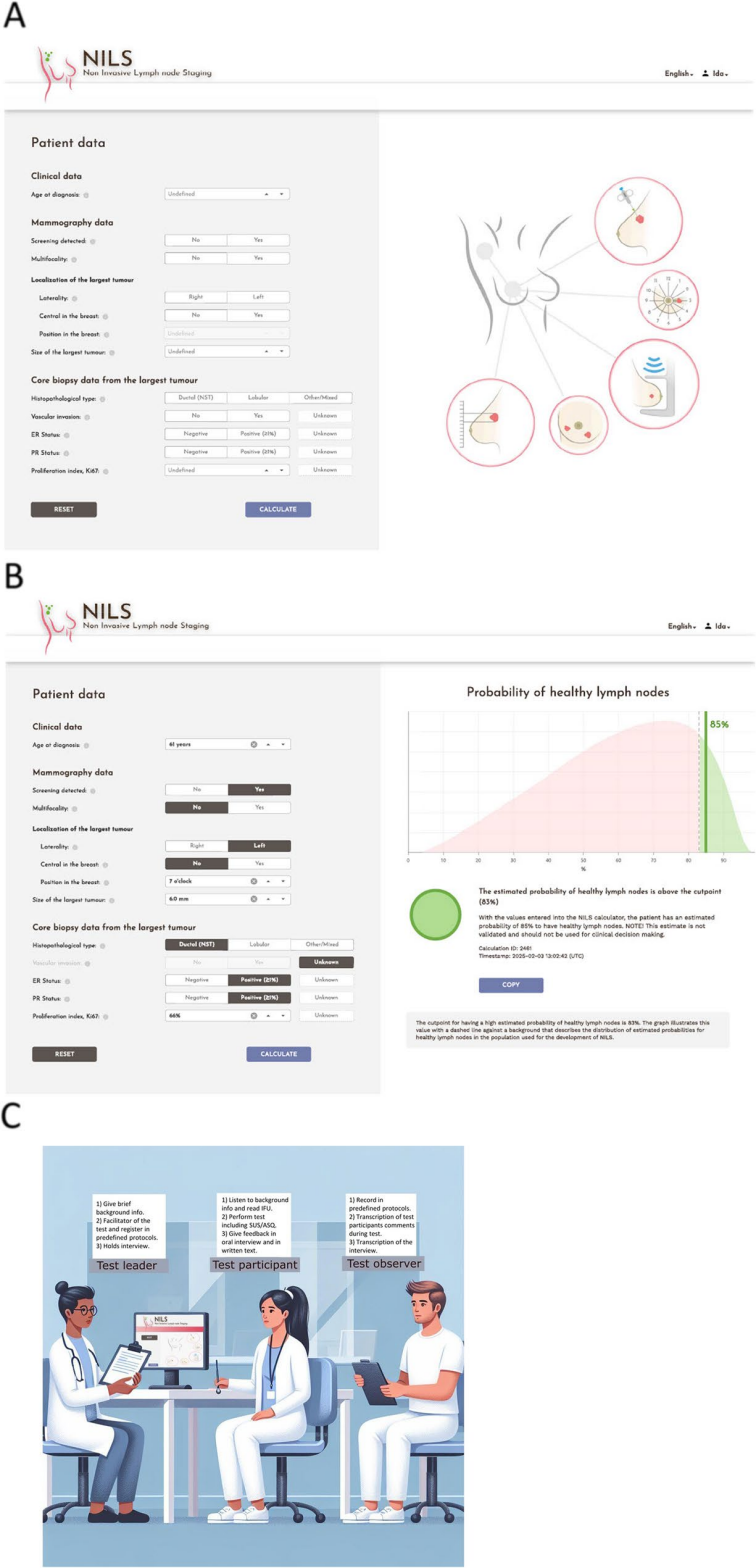
The NILS model webpage intercept as seen in the usability test (**A**) The calculator (**B**) The output of the calculator: histogram and result (**C**) Schematic image of the simulated on-site real-world test sessions. **A** Users can adjust the values for “Age at diagnosis,” “Position in the breast,” and “Proliferation index, Ki67” using up or down arrows. Other fields are completed by clicking on the appropriate options corresponding to the patient details. Each field includes an information button that provides additional information regarding the parameters to be entered. The tumor characteristics “Vascular invasion,” “ER,” “PR,” and “Ki67” can be marked as “Unknown,” and the calculator will still generate a probability of benign axillary lymph nodes. Once all data has been entered, including any “Unknown” values, the user presses the “Calculate” button. The user can reset the calculator to input new values from another patient. **B** Output includes the probability of benign lymph nodes (%) and a preset cutoff based on the false-negative rate for sentinel lymph node biopsy. The text results (including a list of inserted data points) can be copied and pasted onto other documents. The interface logs all the inputs and outputs with individual calculation id and timestamps for identification and traceability. **C** Test sessions were performed with the test leader (first author, a breast surgeon working clinically at the University Hospital test site), a test participant, and an observer in the same room. A computer or laptop already logged into the NILS webpage was provided to the participants to complete the test. The computer was the property of each hospital while the laptop was provided by the test team (Dell Latitude 5430). The observer transcribed all spoken comments made by the test participant. Discrepancies between the test leader’s and observer’s recordings were resolved through discussion and documented as uncertainty in the report. Although the NILS webpage can be accessed via a browser on computers, tablets, or smartphones, only PCs were evaluated, because this is the current standard setup in clinical settings. The image is generated with AI (Microsoft Copilot) followed by substantial modification by the researchers

The calculator interface is divided into two sections: the left side for entering patient and tumor characteristics and the right side for displaying the results, which include the probability of benign lymph nodes and a cut-off point (Fig. 1A and B). After performing the calculation, the results can be copied to the clipboard and pasted onto other documents such as the patient’s medical records. The vendor of the interface logs all inputs and outputs for technical purposes. However, data entered into the model were securely processed on a dedicated research server and were not shared with third parties.

### Study population and selection

The study´s target user population comprised licensed and board-certified medical physicians specializing in general surgery or oncology, representing the intended users. Despite the variations in medical specialties, the participants were considered a unified user group for the study. Given that the NILS model is not yet available on the market, the participants had no prior experience with it, which aligns with the study’s design objectives.

#### Number of test participants

According to the U.S. Food and Drug Administration guidance on Human Factors Validation [16, 17], 20 test participants can identify at least 95% of usability problems, with an average detection rate of 98.5%. Therefore, we considered the number of test participants sufficient. Accessible but diverse study sites were invited to participate in the usability study through oral and e-mail invitations that included a brief explanation of the objectives of the study. Test participants were offered a small symbolic compensation for their participation.

### Study design and setting

We conducted a mixed-method experimental observational study involving user scenarios in a simulated on-site real-world environment at the respective hospitals of the test participants. This approach allowed the test leader and observer to closely monitor and analyze user interactions and behaviors under controlled, yet realistic conditions (Fig. 1C). A pilot session was conducted before the start of the study.

The test participants received a brief introduction to the test session from the test leader to explain how the test would be conducted. A printed copy of the instructions for use (Supplement 1) was provided and they were encouraged to read them before they started the test. They had access to the instructions for use throughout the test, mimicking real-world use of the device. Additionally, the test participants received the test protocol, which included a demographic questionnaire, five simulated cases (Supplement 1), and a response form. They were required to record the calculated probability, interpret the smoothed histogram, and select the appropriate clinical pathway based on the NILS model’s results and other available information. The options included: “Consider omitting SLNB,” “Consider performing SLNB,” “Definitely perform SLNB,” and “NILS cannot assist in making the clinical decision.” Suggested treatment decisions were not evaluated in this study. Lastly, in each session, the overall usability of the device was evaluated through the validated System Usability Scale (SUS), which is one of the most commonly used usability assessment questionnaires [18, 19], and satisfaction was assessed using the After-Scenario Questionnaire (ASQ), in which test participants responded using Likert Scales from 1 to 5 and 1–7, respectively [20]. Written feedback was obtained. Oral interview questions were administered after the interface use if the test leader or observer noticed that the participant had completed a task incorrectly, nearly made an error, or showed use difficulties. Each test session was scheduled for one hour, including the validated questionnaires and oral interview. While the test participants performed each scenario, both the test leader and observer recorded performance on each task in two separate predefined protocols, as: “correct use,” ”use error,” “close call,” or “use difficulty” (Supplement 2). Subsequently, the tasks were evaluated as either a “pass“ or “fail.” Discrepancies between the test leader’s and observers’ recordings were resolved through discussion and documented as uncertainty in the report.

### Summary of previous usability evaluations

A formative evaluation was conducted with six participants who responded to questions after using the calculator, none of whom reported any serious usability issues. However, during testing, concerns were raised regarding the potential for inaccurate data entry or misinterpretation of the results. These concerns prompted enhancements to the user interface, such as clearly marking the position of the tumor in the breast and using a different font color to highlight missing data. Briefly, the most hazardous potential harm to be avoided by a safe device design is a false-estimated indication of benign sentinel lymph nodes. A list of identified potential use errors is provided in Supplement 3.

### Identification and description of critical tasks

Critical tasks were identified after considering previously identified risks in the risk analysis: reset the calculator, enter clinical data, enter mammography data, enter core biopsy data, and identify the appropriate clinical pathway (considering the results of the NILS calculator in combination with all other available information of the case) [21]. The simulated cases were created to ensure that all critical tasks were performed during the test and that the test conditions were sufficiently realistic to represent actual conditions of use [22]. 

### Predefined acceptance criteria for evaluation

Since the NILS model has not yet been released into the market and has limited exposure to intended users, the team anticipated potential usability issues. To assess performance, an acceptance criterion was set at ≥ 90% successful task completion, evaluated on a pass/fail basis for each task.

### Data analysis

Descriptive statistics were calculated for test participants’ characteristics. SPSS Statistics version 28 (IBM Corp., Armonk, NY, USA) was used for all statistical analyses. The SUS score was calculated [23] (mean and median) and interpreted according to the Adjective Rating Scale [24]. In terms of qualitative data, notes taken during test sessions by the test leader and observer were rigorously reviewed during data analysis and systematically categorized to gather comments and suggestions of redesign of the interface.

## Results

In this multisite simulated test session-based usability study with intended users, 20 physicians from four hospitals participated, including 9 (45%) within University Hospitals and 11 (55%) within Regional Hospitals covering three health care regions. Of the twenty participants, 13 (65%) were women and 11 (55%) were < 50 years. The majority were surgeons, comprising 75% (*N* = 15) of the participants, while the remaining 25% (*N* = 5) were oncologists (Table 1).

**Table 1:** Self-reported characteristics of usability testing participants

		*N* (%)
Physician characteristics		Usability testing participants (*N* = 20)
Specialty	Surgeon	15 (75%)
	Oncologist	5 (25%)
Completed years at specialist (years), median (IQR)		9.5 (7.0-–17.3)
Use of any tool/system for prediction in clinical work	Yes	14 (70%)
	No	6 (30%)
- If, yes – which?		*N* = 14, Predict (100%)
Workplace (Hospital)	University Hospital	9 (45%)
	Regional Hospital 1	2 (10%)
	Regional Hospital 2	5 (25%)
	Regional Hospital 3	4 (20%)
Highest academic degree	MD	12 (60%)
	MD + PhD	6 (30%)
	Associate Professor	1 (5%)
	Professor	1 (5%)
Gender	Male	7 (35%)
	Female	13 (65%)
	Other	-
	Do not want to say	-
Age (years), median (IQR)		48.5 (43.3–52.0)

*Abbreviations*: *IQR* interquartile range, *MD* Doctor of Medicine, *PhD* Doctor of Philosophy

### Task completion

The three critical tasks (1–6 data entering points/task) of entering clinical, mammography, and core needle biopsy data, respectively, were all above the predefined acceptance criteria of ≥ 90% and thus defined as passed (Table 2 and Supplement 4). The critical task of finding the reset button was just below the predefined acceptance criteria and was defined as a failure. The task of performing the calculation (i.e., conditional on the accuracy of entered data) was just below the predefined acceptance criteria for the first scenario only.

**Table 2:** Frequency of observed outcome and result, analysis per task

	Description	Observed outcome, N (%)				Result, N (%)	
Scenario		Correct use	Use error	Use difficulty	Close call	Pass	Fail
1	Reset the calculator*	N/A	N/A	N/A	N/A	N/A	N/A
	Enter clinical data	20 (100)	0 (0)	0 (0)	0 (0)	20 (100)	0 (0)
	Enter mammography data	18 (90)	1 (5)	1 (5)	0 (0)	19 (95)	1 (5)
	Enter core biopsy data	18 (90)	2 (10)	0 (0)	0 (0)	18 (90)	2 (10)
	Perform calculation	17 (85)	3 (15)	0 (0)	0 (0)	**17 (85)**	**3 (15)**
	Select the appropriate clinical pathway	16 (80)	2 (10)	1 (5)	1 (5)	18 (90)	2 (10)
2	Reset the calculator	17 (85)	3 (15)	0 (0)	0 (0)	**17 (85)**	**3 (15)**
	Enter clinical data	20 (100)	0 (0)	0 (0)	0 (0)	20 (100)	0 (0)
	Enter mammography data	19 (95)	0 (0)	1 (5)	0 (0)	20 (100)	0 (0)
	Enter core biopsy data	18 (90)	2 (10)	0 (0)	0 (0)	18 (90)	2 (10)
	Perform calculation	18 (90)	2 (10)	0 (0)	0 (0)	18 (90)	2 (10)
	Select the appropriate clinical pathway	19 (95)	1 (5)	0 (0)	0 (0)	19 (95)	1 (5)
3	Reset the calculator	17 (85)	3 (15)	0 (0)	0 (0)	**17 (85)**	**3 (15)**
	Enter clinical data	4 (20)	0 (0)	16 (80)	0 (0)	20 (100)	0 (0)
	Select the appropriate clinical pathway	20 (100)	0 (0)	0 (0)	0 (0)	20 (100)	0 (0)
4	Reset the calculator	17 (85)	3 (15)	0 (0)	0 (0)	**17 (85)**	**3 (15)**
	Enter clinical data	19 (95)	1 (5)	0 (0)	0 (0)	19 (95)	1 (5)
	Enter mammography data	20 (100)	0 (0)	0 (0)	0 (0)	20 (100)	0 (0)
	Enter core biopsy data	19 (95)	1 (5)	0 (0)	0 (0)	19 (95)	1 (5)
	Perform calculation	18 (90)	2 (10)	0 (0)	0 (0)	18 (90)	2 (10)
	Select the appropriate clinical pathway	19 (95)	1 (5)	0 (0)	0 (0)	19 (95)	1 (5)
5	Reset the calculator	17 (85)	3 (15)	0 (0)	0 (0)	**17 (85)**	**3 (15)**
	Enter clinical data	20 (100)	0 (0)	0 (0)	0 (0)	20 (100)	0 (0)
	Enter mammography data	20 (100)	0 (0)	0 (0)	0 (0)	20 (100)	0 (0)
	Enter core biopsy data	18 (90)	2 (10)	0 (0)	0 (0)	18 (90)	2 (10)
	Perform calculation	18 (90)	2 (10)	0 (0)	0 (0)	18 (90)	2 (10)
	Select the appropriate clinical pathway	20 (100)	0 (0)	0 (0)	0 (0)	20 (100)	0 (0)

*N/A: Resetting the calculator was not applicable in scenario 1

Results presented in plain font indicates a task result categorized as “pass” (meeting or exceeding the predefined acceptance criteria of ≥ 90%), whereas result presented in bold font indicates a task result categorized as “fail” (falling below the predefined acceptance criteria of ≥ 90%)

The critical task of selecting the appropriate clinical pathway (based on the calculation results and case information) met or exceeded the predefined acceptance criteria, and was successfully completed for all five scenarios.

### Usability and satisfaction

Test participants found the NILS tool to be highly user-friendly; the SUS score averaged 89.5 and 89.4 points (mean) on a question- and test participant-based analysis, respectively (Supplements 5 and 6). Moreover, the NILS model yielded high overall satisfaction with an averaged mean ASQ score of 6.3 (maximum 7) (Supplement 7).

### Usability domains

The test participants explained their interpretation of the NILS histogram following each case and declared a good understanding of the presented results (Table 3). However, they requested more detailed information regarding what the histogram represented, and how to interpret it in a clinical setting.

**Table 3:** Illustrative comments about the NILS model

Theme	Comment
General impression	“Easy to use with an appealing layout.”
	“Pleasant number of variables.”
	“The results are aligned with my clinical intuition.”
Barriers to the NILS model use	“I am not completely comfortable with the presented histogram and the suggested threshold. I would prefer a recommendation from the calculator regarding omission or not of SLNB.”
	“I’d like a simple explanation of what the imputed values represent and how they will impact the risk estimation.”
Clinical content	“Curious to why tumor grade and HER2-status are not part of the calculator. The inclusion of these parameters would, in my opinion, increase the credibility of the model.”
	“For multifocal tumors, what if the smaller ones have a more worrisome profile? Then I would decide on SLNB depending on these characteristics.”
	“For grade 3 tumors I would always opt for SLNB. That is what my clinical experience tells me.”
	“It seems more intuitive to be presented the risk of metastatic lymph nodes.”
	“I’d prefer an overview of contraindications for the NILS calculator, such as neoadjuvant treatment.”
Interpretation of the histogram	“The risk is too high for metastatic lymph node to safely omit SNLB, the probability for benign lymph nodes is below the threshold value, can´t omit SLNB.”
	“The calculated value is above the threshold value – probably benign lymph nodes. There is a low risk for metastatic lymph nodes, the consideration to omit SNLB can be discussed with the patient.”
	“The probability of benign lymph nodes is just above the threshold to consider omitting SLNB. Since the calculated value is very close to the threshold, I would have done SLNB.”
	“The calculated probability is clearly below threshold value; it is not appropriate to omit SLNB.”

Comments are paraphrased to preserve the original posters’ anonymity. Translation from the original language spoken at the sessions (Swedish) to English by the researchers

Several redesign elements were identified, addressing both technical components and educational/informational challenges (Table 4). A recurring concern was the exclusion of the histological tumor grade and human epidermal growth factor 2 (HER2) status, which are clinically important for recommending neoadjuvant or adjuvant treatments. The term “multifocality” caused some hesitation, as participants were concerned that smaller tumors, excluded from calculations, might have less favorable characteristics than the largest tumor, requiring additional consideration. The reset button was difficult to locate because of its inconspicuous black color and placement at the bottom of the calculator, which became hidden when users viewed the resulting histogram at the top. In addition, users can accidentally scroll within the last cell of an entered value, often altering the Ki67 value. This led one participant to enter an incorrect value, resulting in a miscalculated probability of benign lymph nodes. Furthermore, the information buttons were often missed by the participants and hence were rarely used. The implemented interface modifications are detailed in Table 4 and further illustrated in Supplement 8.

**Table 4:** Major design requirements derived from the usability study with intended users

Domian	Source	Issue	Requirements	Implemented Alterations
Tool Operation	It is possible to enter patients’ age outside of the accepted range.	When the age is outside the accepted range the cell becomes yellow, however, still it is possible to enter a value. If notice is not taken, unnecessary work is done inserting all other variables. It will, however, not be possible to make a NILS calculation.	A clear notice that inserted value is out of range/not possible to insert value out of range.	The cell is highlighted in red and an explanatory error message is shown underneath the field to the user.
	The NILS calculator is applicable to T1-2 breast cancer. However, in the calculator it is possible to enter a tumor size of up to 90 mm.	If notice is not taken that tumor size is out of range, unnecessary work is done inserting all other variables. It will, however, not be possible to make a NILS calculation.	A clear notice that inserted value is out of range/not possible to insert value out of range.	The cell is highlighted in red and an explanatory error message is shown underneath the field to the user.
	The placement of the reset button is counterintuitive in the web interface, making it difficult to use correctly.	The reset button is placed at the bottom of the calculator. When making the calculation the user is directed automatically to the histogram, which is displayed in the upper part of the calculator and the reset button then falls out of sight.	It would enable correct use to place the reset button at the top of the calculator, in the eyeline of the user when entering data for a subsequent case. Further, the black colored reset button does not draw attention, so another color is preferable.	The reset button was moved to the top of the interface and highlighted using more prominent colors (blue and white), together with the addition of a reset icon.
	When scrolling down the webpage to reach the calculate button, it is possible to accidentally scroll within the last cell, most commonly the Ki67 value for the largest invasive tumor.	The scrolling can alter the inserted value and if not identified and corrected cause the calculated value to be wrong.	Ensure that scrolling cannot alter an entered value.	The cell component was redesigned so that scrolling no longer alters entered values.
	Uncertainty about how to handle multifocality and central tumor, the information provided by the calculator was often overlooked.	When struggling with the decision on which tumor variables to enter in case of multifocality and how a central tumor is defined, the information button was seldom identified.	To enhance the ability to find the information button, it needs to be more clearly marked.	The information button was repositioned to enhance visibility and highlighted using more prominent colors (blue and white). A clearer link to the Instructions for Use was added. When multifocality is selected, the heading updates dynamically to indicate that the entered information refers to the largest tumor.
Clinical Safety Concerns	In the histogram, the cut-off is marked with a stretched line and the cut-off value is presented in text below. The calculated percentages are marked as a filled line with calculated value presented at the top within the histogram figure.	The cut-off value can be misinterpreted as the calculated value if the y-axis and the text below only are viewed.	The cut-off value should also be visualized with a number in addition to the line in the histogram. Moreover, in the text below the histogram, the resulting percentage and not the cut-off value should be emphasized.	The cut‑off value was added to the histogram, and the legend was updated to emphasize the resulting percentages rather than the cut‑off value.
	The histogram is presented without declaring what the x-axis and y-axis represent.	For correct interpretation of the presented histogram/result, proper labelling is warranted.	Add the following descriptions for the x- and y-axes, respectively: “Estimated probability of benign lymph nodes in the axilla” and “Fraction of patients in the cohort used for model development”.	The axis descriptions were revised accordingly to improve clarity.
	The information on applicable use population, e.g. exclusion of neoadjuvant-treated patients, is not easily found when working with the NILS calculator.	If disclaimers are not noted, the NILS model can be used by mistake on the wrong patient population.	In the current version, “Disclaimers” can be found in the ribbon at the top of the calculator interface. It is accessible only by navigating to an adjacent webpage. To improve usability, consider including a heading within the calculator page stating “The NILS calculator is not intended for use in every scenario.”, followed by a link to “Instructions for Use.”	A clearer link to the Instructions for Use was added.

## Discussion

Using an experimental mixed-methods observational study design with user scenarios in a simulated on-site environment, we found broad support among licensed medical physicians for the NILS model. This tool provides risk estimates for benign sentinel lymph node status, aiding attending physicians in deciding whether or not SLNB can be abstained. The test participants identified several usability concerns, as well as barriers and facilitators to the implementation and widespread use of the NILS model. These insights have prompted future iterative design modifications.

### Principal findings

Many new insights were gained during this usability study. Several aspects of the existing interface posed challenges for the test participants. These included difficulties in locating the reset and information buttons, as well as technical issues, such as the potential to enter values outside the applicable ranges (e.g., for age and tumor size). In addition, the risk of unintentionally altering the entered value during scrolling was detected. Furthermore, a learning curve was evident. Data entry and calculations were the most challenging in scenario 1 and became progressively easier in subsequent scenarios. This is reflected in the only failure of the “perform calculation” task, which occurred during the first scenario.

The accomplished SUS score, which is recommended to be regarded as a percentile and not as a percentage, was categorized as “excellent” (score > 85). The slight discrepancy between the per-question and per-participant analyses was due to one slightly skeptical participant who was dissatisfied with the model’s role as a clinical support tool. This participant specifically sought a definitive clinical decision regarding whether to perform SLNB. A high ASQ score indicated that the test participants found it easy to complete the tasks in the NILS calculator, were satisfied with the time taken to complete the tasks and found the support information adequate.

### Comparison to similar devices

Benchmark devices for the NILS model have not been identified. A tool known as Predict, which estimates the survival effect of selected adjuvant therapies for breast cancer, has been widely utilized by both patients and healthcare professionals [25]. A completed usability study on Predict has provided six key recommendations that have been considered in the planning of this NILS model usability study.

As for the NILS model, which is designed to facilitate decision-making, Predict serves as an adjunct to recommended adjuvant therapies according to established guidelines, rather than as a stand-alone decision tool. However, a significant distinction between the NILS model and Predict lies in their functionalities. Unlike Predict, which compares oncological outcomes and the advantages and disadvantages of different treatment options for patients with specific characteristics, the NILS model aids in the decision to abstain from or perform SLNB.

### Clinical contextualization

Comprehending the underlying rationale of the model predictions is essential for building trust in the results calculated using the NILS model. Therefore, we suggest, in accordance with Farmer et al., [25] that the provision of contextual information making the calculator´s results useful in their clinical setting is important for a positive attitude of the intended users, that is, health care professionals. In this study, we identified the need for enhanced assistance in interpreting histograms, including explaining the rationale behind the cutoff value. There was also a request for a comprehensive explanation of the inclusion and exclusion criteria for the relevant patient population to deepen knowledge of why certain variables, such as HER2 status and histological tumor grade, were not included in the NILS model.

### Technological barriers

Mistrust and unfamiliarity with machine learning models are frequently identified as significant barriers to their integration into clinical practice [26]. Although interpreting the resulting histogram was challenging, none of the test participants showed technological mistrust towards the machine learning model behind the NILS calculator. This trust in the model is anticipated to be generalizable to intended users, given the careful consideration of the test participants’ representativeness of future intended users.

### Strengths and limitations

The study was rigorously planned and executed through thorough data control. All endpoints and acceptance criteria were registered before study initiation. Conducted with the intended users, including clinical surgeons and oncologists with representative post-specialist clinical experience, the study was conducted in a simulated on-site real-world physical and digital environment within three healthcare regions. This approach enhanced the generalizability of the results to actual clinical practice, primarily within Sweden and to comparable health care systems, but not necessarily to less digitally integrated settings. Having both a test leader and observer ensured strict adherence to the study protocol, guaranteeing objectivity throughout the process.

There were no requirements for user training before the device was used. However, in a real-world scenario, users might take varying amounts of time to read the “Instruction for Users”; some might read it thoroughly from front to back, while others might only glance at it before starting to use the device. Moreover, we hypothesized that the NILS model could be effectively utilized in a multidisciplinary conference setting, where physicians from various specialties discuss and formulate treatment plans for specific patients, considering the available data not included in the NILS model. Although this particular clinical scenario was not replicated in this usability study, we anticipate that similar criticisms and evaluations will emerge.

### Future perspectives

Several key redesign elements were identified, encompassing technical aspects and educational and informational issues. Collectively, these changes will enhance the usability and workflow, thereby preparing the NILS model for clinical use as a decision-support tool. The proposed improvements have been implemented and evaluated with an independent group of test participants (Supplement 8). In a post-market setting, the user interface will be iteratively updated and evaluated as needed using information gained from the post-market process.

## Conclusion

This qualitative premarket usability study of the NILS model, conducted through test sessions with simulated clinical cases, involved intended users, that is, surgeons and oncologists, in a real-world clinical setting. This study identified the key usability factors, barriers, and facilitators influencing implementation. Physicians found the NILS model intuitive and valued its presented risk estimates for benign axillary lymph node status in their decision to perform or abstain from SLNB. These findings have directly informed the refinement of the NILS model, ensuring improved usability and clinical integration.

## Supplementary Information

Supplementary Material 1. Usability study protocols.

Supplementary Material 2. Definitions applied during the usability testing for observed outcomes for each predefined task.

Supplementary Material 3. Specifications of potential use errors that can be avoided through safe devise design.

Supplementary Material 4. Frequency of observed outcome and result, analysis per task description.

Supplementary Material 5. The System Usability Scale (SUS) to assess participants’ levels of agreement with the overall usability of the system. Test participants N=20 (results per question).

Supplementary Material 6. The System Usability Scale (SUS) to assess participants’ level of agreement with the overall usability of the system (results per test participant).

Supplementary Material 7. After-Scenario Questionnaire (ASQ) to assess the participants’ level of satisfaction with the NILS model. Test participants N=20 (results per question).

Supplementary Material 8. Implemented alterations of the interface of the NILS model and results of repeated usability testing. Test participants N=6.

## Data Availability

The raw datasets are available from the corresponding author on reasonable request.

## Abbreviations

ASQ: After Scenario Questionnaire
BCS: Breast-conserving surgery
HER2: Human epidermal growth factor receptor 2
NILS: Noninvasive lymph node staging
SLNB: Sentinel lymph node biopsy
SUS: System Usability Scale
