# Supplementary material for: Human factors validation study of an artificial neural network‑based preoperative decision‑support tool for noninvasive lymph node staging (NILS) in women with primary breast cancer (ISRCTN99301435)
Source: BMC Cancer. 2026 May 28;26:691. doi: 10.1186/s12885-026-16161-5 (PMC13221748; doi:10.1186/s12885-026-16161-5)
Supplement: Supplementary file 1 — Supplementary Material 1. Usability study protocols. [file 12885_2026_16161_MOESM1_ESM.pdf]

Supplementary Material 1. Usability Study Protocols

A. Instruction for Use
B. Instruction for Test Participant
C. Instruction for Test Leader
D. Instruction for Observer

A

Instructions for use NILS

1 About NILS

NILS (Non-Invasive Lymph Node Status) is a web-based tool that calculates the probability of a healthy axilla in women with primary breast cancer. By analysing entered patient data and tumour characteristics, NILS enables a non-invasive assessment of lymph node status in the axilla. The tool serves as a decision support and facilitates the decision of whether sentinel node biopsy (SLNB) should be performed in patients undergoing primary breast cancer surgery.

Note that NILS is intended to be used in the context of clinical and usability studies. During this validation phase, the tool is not intended to provide specific medical advice regarding the treatment of breast cancer. The results from the web-based calculator will therefore not be shared with either the patient or the attending physician during this validation phase. Please note that NILS is currently not CE-marked and shall not be used in clinical decision-making.

1.1 Instructions for use

The instructions for use (this document) must be read before using the product.

1.2 General information

**Name of the device**	NILS
**UDI-DI**	*Not yet assigned*
**UDI-PI**	*Not yet assigned*
**Intended use**	NILS is a web-based calculator that uses artificial intelligence to calculate the probability that the sentinel node(s) are healthy (benign) in women with a clinically node-negative axilla who are scheduled for primary surgery for invasive breast cancer.<br><br>NILS is intended to be used preoperatively by attending physicians, surgeons and oncologists, to facilitate risk-benefit analysis and patient stratification prior to decisions on performing SLNB. In women with a high calculated probability of having benign axillary lymph nodes, NILS should be used as a complementary clinical decision support and not as a stand-alone tool for the decision to forgo SLNB.
**Warnings and precautions**	No warnings or precautions have been identified.
**Patient population**	Women aged 24 to 100 years, who are planned for primary surgery due to invasive breast cancer and are clinically axillary node negative.
**Version of NILS**	R1a
**Indications**	Primary invasive breast cancer.
**Contraindications**	The device is not intended for male patients.<br>The device is not intended for patients scheduled for neoadjuvant therapy.

	The device is not intended for patients that have previously undergone ipsilateral surgery in the breast and/or axillary surgery due to invasive breast cancer or ductal carcinoma in situ DCIS).<br>The device is not intended for patients with biopsy verified ductal carcinoma in situ (DCIS) without primary invasive breast cancer.<br>The device is not intended for patients with clinical axillary node positive disease (palpable or ultrasound positive).<br>The device is not intended for patients with biopsy verified axillary lymph node metastasis.<br>The device is not intended for patients with T3/T4 tumours (tumours larger than 5 cm).
**Intended user**	Professional use only: attending physician
**Limits of accuracy**	The probability (percentage without decimals) of healthy axillary lymph nodes is calculated using a cutoff chosen based on the same accepted false-negative rate as for SLNB (<10%).
**Specification of the population the AI algorithm is trained on**	Dihge et al. BMC Cancer 2019.
**Residual risks and side-effects**	No residual risks or side-effects have been identified. The risk analysis will be updated after the usability study.
**Clinical benefit**	Act as a decision support to identify patients at low risk of axillary lymph node metastases, and thereby contribute to the abstaining of SLNB and subsequently reduce the negative consequences of the procedure.

1.3 Technical description

To use NILS, a stable internet connection is required. The website is adapted for use with a tablet or mobile phone.

1.4 Address

NILS is developed by Region Skåne.

Region Skåne
291 89 Kristianstad

1.5 Explanation of symbols

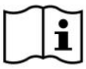

Instructions for use (this document) shall be read before using the device.

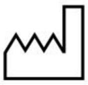

Date of the last update of the device.

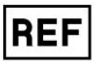

Version number of the device.

2 How to use NILS

1. Open the browser and go to https://nils.cec.lu.se/
2. Click ’Log in’.
3. Enter your personal username and password and click ’Log in’.
4. Choose ’To calculator’ to get to the calculator.

5. Enter patient and tumour information in the intended fields (see details below).

   Note that you actively have to choose a value in all fields. However in some fields the value 'Unkown' is an option if the value is not available.

Variables	Allowed values	Comments
Age at diagnosis (years)	Integer: 24 – 92	For example, a person is considered to be 54 years old until the day they turn 55 years.
Screening detected	No / Yes	-
Multifocality	No / Yes	-
**Placement of the largest tumour**		
Laterality	Right / Left	Refers to the patient´s right / left breast
Central in the breast	No / Yes	-
Position in the breast	Integer: 1–12	This option can only be chosen if 'Central in the breast = No' has been selected in the previous step.<br><br>Clock 1 to 12 is used to localise the tumours position in the breast from the clinician's perspective when observing the patient.
Size of the largest tumour (mm)	0.5 – 50.0	Note! only T1-T2 (50 mm or smaller)
**Core biopsy data**		
Histopathological type	Ductal (NST)<br>Lobular<br>Other/Mixed	-
Vascular invasion	No / Yes / Unknown	-
ER status	Negative<br>Positive (≥ 1%)<br>Unknown	Note – the threshold of the calculator is set to ≥ 1%, as opposed to the threshold of ≥ 10% that is used in Swedish clinical practice.
PR status	Negative<br>Positive (≥ 1%)<br>Unknown	Note – the threshold of the calculator is set to ≥ 1%, as opposed to the threshold of ≥ 10% that is used in Swedish clinical practice.
Ki67 (%)	0 – 100	-

3 Interpretation of results

The calculated probabilities, along with whether the result fall below or exceed the cutoff, can serve as a foundation for discussions with the patient regarding her individual likelihood of having healthy lymph nodes. The results can help clarify the risk profile and support informed decisions about the necessity of SLNB.

The NILS model is designed to handle missing (lympho)vascular invasion (LVI) information by imputing this value. In the validation study (Dihge et al., Front Oncol 2024), the model reached an AUC of 0.735 (95% CI, 0.704–0.764) when information on other variables was available. Furthermore, the model is adapted to perform predictions when information on

ER status, PR status, and/or Ki67 is missing, in which the predictive performance of healthy lymph nodes only marginally decreased to AUC = 0.718 (95% CI, 0.687–0.748).

3.1 The histogram

Red colour indicates that the estimated probability of healthy axillary lymph nodes is **lower** than the cutoff.

Green colour indicates that the estimated probability of healthy lymph nodes is **higher** than the cutoff.

4 Support and contact

If you need help, contact Professor Lisa Rydén at lisa.ryden@med.lu.se

5 References

Dihge L, Ohlsson M, Edén P, Bendahl PO, Rydén L. Artificial neural network models to predict nodal status in clinically node-negative breast cancer. *BMC Cancer*. 2019 Jun 21;19(1):610. doi: 10.1186/s12885-019-5827-6. PMID: 31226956; PMCID: PMC6588854.

Skarping I, Ellbrant J, Dihge L, Ohlsson M, Huss L, Bendahl PO, Rydén L. Retrospective validation study of an artificial neural network-based preoperative decision-support tool for noninvasive lymph node staging (NILS) in women with primary breast cancer (ISRCTN14341750). *BMC Cancer*. 2024 Jan 16;24(1):86. doi: 10.1186/s12885-024-11854-1. PMID: 38229058; PMCID: PMC10790472.

Hjärtström M, Dihge L, Bendahl PO, Skarping I, Ellbrant J, Ohlsson M, Rydén L. Noninvasive Staging of Lymph Node Status in Breast Cancer Using Machine Learning: External Validation and Further Model Development. *JMIR Cancer*. 2023 Nov 20;9:e46474. doi: 10.2196/46474. PMID: 37983068; PMCID: PMC10696498.

Appendix B1: Test instructions for participants – English

**ID (to be filled in by test leader)**	

You will be asked to perform five scenarios, with five different patient cases. The cases are presented below. After calculating each case using NILS, fill in the appropriate clinical pathway based on the calculation and other available information in the table below. For each new case you have to clear the interface before entering the new information. It is possible that not all cases can be calculated in NILS. Make sure that you finish each case before you start the next case. If a case cannot be calculated, the column "NILS cannot assist in making the clinical decision" shall be marked in the result table below. You may not ask the test leader for assistance during the test, however you may ask organizational questions related to how the test session will be conducted. You have the instructions for use available during the whole session.

Please note that we want to evaluate the usability of the calculator, not the clinical decision-making!

Please fill in the information below.

**Initials:**	
**Date:**	
**Speciality:**	☐ Surgeon<br>☐ Oncologist<br>☐ Experience as a specialist (years):
**Hospital you work at:**	☐ SUS<br>☐ Växjö<br>☐ Kristianstad<br>☐ Karlskrona<br>☐ Other:
**Academic degree:**	☐ MD<br>☐ PhD
**Age:**	
**Gender:**	☐ Female<br>☐ Male<br>☐ Other<br>☐ Do not want to say
**Do you use any tool/system for prediction in your work today? If yes, which?**	☐ Yes<br>☐ No<br>If yes, which:

Cases

Case 1

A previously healthy 61-year-old woman with a screening detected lesion of one tumour in the left breast. The clinical mammography and ultrasound state a single 11 mm retromammillary tumour (approximately 1 cm from the mammilla) in the left breast. Ultrasonography revealed no suspicious findings in the axilla. Core needle biopsy results reported invasive NST; histologic grade 2; ER 98%; PgR 98%; HER2-negative and Ki67 77%. Vascular invasion was neither confirmed nor denied. Clinical examination of the left breast and axilla revealed no abnormalities.

Question	Your answer
What percentage did the calculation result in?	
How should the presented histogram be interpreted for Case 1?	

Please fill in the clinical pathway that you find most appropriate based on the information you have received and the NILS calculation. Note that several pathways can be appropriate.

	Consider omitting SLNB	Consider performing SLNB	Definitely perform SLNB	NILS cannot assist in making the clinical decision
**Case 1**				

Case 2

An 83-year-old woman with no previous history of malignancy. Pharmacologically treated hypertension and hyperlipidemia. Presents with a self-detected palpable mass in the left breast. The clinical mammography and ultrasound state a single 18 mm tumour at 6 o'clock, 3 cm from the mammilla in the left breast. Ultrasonography revealed no suspicious findings in the axilla. Core needle biopsy results reported invasive mixed ductulobular; histologic grade 3; ER 78%; PgR 91%; HER2-negative and Ki67 18%. Vascular invasion was neither confirmed nor denied. The tumour was confirmed palpable upon clinical examination of the left breast and the examination of the axilla revealed no abnormalities.

Question	Your answer
What percentage did the calculation result in?	
How should the presented histogram be interpreted for Case 2?	

Please fill in the clinical pathway that you find most appropriate based on the information you have received and the NILS calculation. Note that several pathways can be appropriate.

	Consider omitting SLNB	Consider performing SLNB	Definitely perform SLNB	NILS cannot assist in making the clinical decision
**Case 2**				

Case 3

A 22-year-old woman with a self-detected palpable mass in the right breast. The clinical mammography and ultrasound state a single 50 mm tumour at 6 o'clock, 4 cm from the mammilla in the right breast. Ultrasonography revealed no suspicious findings in the axilla. Core needle biopsy results reported invasive NST; histologic grade 3; ER <1%; PgR <1%; HER2-negative and Ki67 76%. Vascular invasion was neither confirmed nor denied. The tumour was confirmed palpable upon clinical examination of the right breast and the examination of the axilla revealed no abnormalities.

Question	Your answer
What percentage did the calculation result in?	
How should the presented histogram be interpreted for Case 3?	

Please fill in the clinical pathway that you find most appropriate based on the information you have received and the NILS calculation. Note that several pathways can be appropriate.

	Consider omitting SLNB	Consider performing SLNB	Definitely perform SLNB	NILS cannot assist in making the clinical decision
Case 3				

Case 4

A previously healthy 68-year-old woman with screening detected findings in the left breast. The clinical mammography and ultrasound state a single 12 mm tumour at 9 o'clock, 5 cm from the mammilla in the left breast. Ultrasonography revealed no suspicious findings in the axilla. Core needle biopsy results reported invasive NST; histologic grade 3; ER 89%; PgR 91%; HER2-negative and Ki67 8%. Vascular invasion was neither confirmed nor denied. Clinical examination of the left breast and axilla revealed no abnormalities.

Question	Your answer
What percentage did the calculation result in?	
How should the presented histogram be interpreted for Case 4?	

Please fill in the clinical pathway that you find most appropriate based on the information you have received and the NILS calculation. Note that several pathways can be appropriate.

	Consider omitting SLNB	Consider performing SLNB	Definitely perform SLNB	NILS cannot assist in making the clinical decision
Case 4				

Case 5

A 43-year-old woman with screening detected findings of bifocal breast tumour in the right breast. The clinical mammography and ultrasound states: 1) a 15 mm tumour at 10 o'clock, 4 cm from the mammilla in the right breast, and 2) 12 mm tumour at 7 o'clock, 3 cm from the mammilla in the right breast. Ultrasonography revealed no suspicious findings in the axilla. Core needle biopsy results reported: "Tumour 1." Invasive NST; histologic grade 2; ER 99%; PgR 99%; HER2-negative and Ki67 76%. Vascular invasion was neither confirmed nor denied. "Tumour 2." Invasive NST, histologic grade 2; ER 98%; PgR 98%; HER2-neg Ki67 71%. Vascular invasion was neither confirmed nor denied. Upon clinical examination, the physician found "tumour 1" palpable in the right breast. Clinical examination of the right axilla revealed no abnormalities.

Question	Your answer
What percentage did the calculation result in?	
How should the presented histogram be interpreted for Case 5?	

Please fill in the clinical pathway that you find most appropriate based on the information you have received and the NILS calculation. Note that several pathways can be appropriate.

	Consider omitting SLNB	Consider performing SLNB	Definitely perform SLNB	NILS cannot assist in making the clinical decision
Case 5				

Please answer the questions in the table below.

This questionnaire gives you an opportunity to tell us your reactions to the system you used. Your responses will help us understand what aspects of the system you are particularly concerned about and the aspects that satisfy you. To as great a degree as possible, think about all the tasks that you have done with the system while you answer these questions. Please read each statement and indicate how strongly you agree or disagree with the statement by checking the box that corresponds best to how you feel. Thank you!

		Strongly Disagree 1	2	3	4	Strongly Agree 5
1	I think that I would like to use this system frequently					
2	I found the system unnecessarily complex					
3	I thought the system was easy to use					
4	I think that I would need the support of a technical person to be able to use this system					
5	I found the various functions in this system were well integrated					
6	I thought there was too much inconsistency in this system					
7	I would imagine that most people would learn to use this system very quickly					
8	I found the system very awkward to use					
9	I felt very confident using the system					
10	I needed to learn a lot of things before I could get going with this system					

1. Overall, I am satisfied with the ease of completing the tasks in this scenario.

Strongly disagree ←→ Strongly agree

1 2 3 4 5 6 7 N/A

Comments:

2. Overall, I am satisfied with the amount of time it took to complete the tasks in this scenario.

Strongly disagree ←→ Strongly agree

1 2 3 4 5 6 7 N/A

Comments:

3. Overall, I am satisfied with the support information (on-line help, documentation) when completing the tasks.

Strongly disagree ←→ Strongly agree

1 2 3 4 5 6 7

Comments: N/A

Do you have any other feedback?

Thank you very much for taking the time to participate in this usability study of NILS!

Appendix A: Instructions and test protocol for test leader

Section 1: Instructions for test leader

Prepare the session by printing a sufficient amount of the IFU, test instructions for participants, and test protocols for the test leader. Ensure that a computer is set up, have a secure internet connection, and is logged into NILS.

When the participant has arrived, greet them and present how the test will be conducted. A brief introduction to NILS may be held, but make sure not to go into details. Explain that they will have the IFU available on paper throughout the whole test.

During the test, the test leader cannot assist or answer questions regarding the use of the product. The test leader may answer questions about the test. The test leader may abort the test if needed, if so, the test shall be marked as failed.

Fill in the test protocol below during the test.

Term	Definition
Correct use	The user completes the task correctly.
Close call	Close calls are instances in which a user has difficulty or makes a use error that could result in harm, but the user takes an action to “recover” and prevents the harm from occurring. Close calls should be recorded when they are observed and discussed with the test participants after they have completed all the use scenarios. In addition, repeated attempts to complete a task and apparent confusion could indicate potential use error and therefore should also be collected as observational data and discussed during the interviews with test participants.
Use error	User action or lack of action that was different from that expected by the manufacturer and caused a result that:<br>(1) was different from the result expected by the user and<br>(2) was not caused solely by device failure and<br>(3) did or could result in harm.
Use difficulty	Use difficulty is when a user seems to struggle to perform a task. This may be observed in different ways: the user may pause to read the manual for a longer time, or a task may take longer time than expected (longer than other tasks) to complete. It is also possible that the participant verbally expresses that a task is difficult. Use difficulty may lead to use errors.

Section 2: Description of test conditions

A computer with an open browser already logged into NILS is available. The participants have received the IFU and test instructions for participants printed on paper. The participant shall fill in the results in the test instructions for participants.

Section 3: Test and user identification

Test date and time:	
Test participant ID:	
Test site:	
Test leader:	
Observer:	

Section 4: Test scenarios

Scenario S1 – Case 1

Task ID	Task description and expected outcome	Observed outcome	Problem	Result	Notes / quotes
S1-1	Enter clinical data.<br>Age: **61**	☐ Correct use<br>☐ Use error<br>☐ Close call<br>☐ Use difficulty		☐ Pass<br>☐ Fail	
S1-2	Enter mammography data<br>Screening detected: **Yes**<br>Multifocality: **No**<br>Laterality: **Left**<br>Central in the breast: **Yes**<br>Position in the breast: **N/A**<br>Size of the largest tumour: **11 mm**	☐ Correct use<br>☐ Use error<br>☐ Close call<br>☐ Use difficulty		☐ Pass<br>☐ Fail	
S1-3	Enter core biopsy data<br>Histopathological type: **Ductal (NST)**<br>Vascular invasion: **Unknown**<br>ER status: **98%**<br>PR status: **98%**<br>Ki67: **77%**	☐ Correct use<br>☐ Use error<br>☐ Close call<br>☐ Use difficulty		☐ Pass<br>☐ Fail	
S1-4	Perform calculation<br>Result is presented with **64 %** probability of healthy lymph nodes.	☐ Correct use<br>☐ Use error<br>☐ Close call<br>☐ Use difficulty		☐ Pass<br>☐ Fail	
S1-5	**Definitely perform SLNB**	☐ Correct use<br>☐ Use error<br>☐ Close call<br>☐ Use difficulty		☐ Pass<br>☐ Fail	

Scenario S2 – Case 2

**Task ID**	**Task description and expected outcome**	**Observed outcome**	**Problem**	**Result**	**Notes / quotes**
S2-1	Reset the calculator	☐ Correct use<br>☐ Use error<br>☐ Close call<br>☐ Use difficulty		☐ Pass<br>☐ Fail	
S2-2	Enter clinical data<br>Age: **83**	☐ Correct use<br>☐ Use error<br>☐ Close call<br>☐ Use difficulty		☐ Pass<br>☐ Fail	
S2-3	Enter mammography data<br>Screening detected: **No**<br>Multifocality: **No**<br>Laterality: **Left**<br>Central in the breast: **No**<br>Position in the breast: **6 o´clock**<br>Size of the largest tumour: **18 mm**	☐ Correct use<br>☐ Use error<br>☐ Close call<br>☐ Use difficulty		☐ Pass<br>☐ Fail	
S2-4	Enter core biopsy data<br>Histopathological type: **Other/mixed**<br>Vascular invasion: **Unknown**<br>ER status: **78%**<br>PR status: **91%**<br>Ki67: **18%**	☐ Correct use<br>☐ Use error<br>☐ Close call<br>☐ Use difficulty		☐ Pass<br>☐ Fail	
S2-5	Perform calculation<br>Result is presented with **87 %** probability of healthy lymph nodes.	☐ Correct use<br>☐ Use error<br>☐ Close call<br>☐ Use difficulty		☐ Pass<br>☐ Fail	
S2-6	**Consider omitting SLNB**	☐ Correct use<br>☐ Use error<br>☐ Close call<br>☐ Use difficulty		☐ Pass<br>☐ Fail	

Scenario S3 – Case 3

**Task ID**	**Task description and expected outcome**	**Observed outcome**	**Problem**	**Result**	**Notes / quotes**
S3-1	Reset the calculator	☐ Correct use<br>☐ Use error<br>☐ Close call<br>☐ Use difficulty		☐ Pass<br>☐ Fail	
S3-2	Enter clinical data<br>Age: **22**<br>Limitation by the calculator, calculation cannot be performed.	☐ Correct use<br>☐ Use error<br>☐ Close call<br>☐ Use difficulty		☐ Pass<br>☐ Fail	
S3-3	**NILS cannot assist in making the clinical decision**	☐ Correct use<br>☐ Use error<br>☐ Close call<br>☐ Use difficulty		☐ Pass<br>☐ Fail	

Scenario S4 – Case 4

Task ID	Task description and expected outcome	Observed outcome	Problem	Result	Notes / quotes
S4-1	Reset the calculator	☐ Correct use<br>☐ Use error<br>☐ Close call<br>☐ Use difficulty		☐ Pass<br>☐ Fail	
S4-2	Enter clinical data<br>Age: **68**	☐ Correct use<br>☐ Use error<br>☐ Close call<br>☐ Use difficulty		☐ Pass<br>☐ Fail	
S4-3	Enter mammography data<br>Screening detected: **Yes**<br>Multifocality: **No**<br>Laterality: **Left**<br>Central in the breast: **No**<br>Position in the breast: **9 o´clock**<br>Size of the largest tumour: **12 mm**	☐ Correct use<br>☐ Use error<br>☐ Close call<br>☐ Use difficulty		☐ Pass<br>☐ Fail	
S4-4	Enter core biopsy data<br>Histopathological type: **Ductal (NST)**<br>Vascular invasion: **Unknown**<br>ER status: **89%**<br>PR status: **91%**<br>Ki67: **8%**	☐ Correct use<br>☐ Use error<br>☐ Close call<br>☐ Use difficulty		☐ Pass<br>☐ Fail	
S4-5	Perform calculation<br>Result is presented with **84%** probability of healthy lymph nodes.	☐ Correct use<br>☐ Use error<br>☐ Close call<br>☐ Use difficulty		☐ Pass<br>☐ Fail	
S4-6	**Consider performing SLNB**<br>**Consider omitting SLNB**	☐ Correct use<br>☐ Use error<br>☐ Close call<br>☐ Use difficulty		☐ Pass<br>☐ Fail	

Scenario S5 – Case 5

Task ID	Task description and expected outcome	Observed outcome	Problem	Result	Notes / quotes
S5-1	Reset the calculator	☐ Correct use<br>☐ Use error<br>☐ Close call<br>☐ Use difficulty		☐ Pass<br>☐ Fail	
S5-2	Enter clinical data<br>Age: **43**	☐ Correct use<br>☐ Use error<br>☐ Close call<br>☐ Use difficulty		☐ Pass<br>☐ Fail	
S5-3	Enter mammography data<br>Screening detected: **Yes**<br>Multifocality: **Yes**<br>Laterality: **Right**<br>Central in the breast: **No**<br>Position in the breast: **10 o´clock**<br>Size of the largest tumour: **15 mm**	☐ Correct use<br>☐ Use error<br>☐ Close call<br>☐ Use difficulty		☐ Pass<br>☐ Fail	
S5-4	Enter core biopsy data<br>Histopathological type: **Ductal (NST)**<br>Vascular invasion: **Unknown**<br>ER status: **99%**<br>PR status: **99%**<br>Ki67: **76%**	☐ Correct use<br>☐ Use error<br>☐ Close call<br>☐ Use difficulty		☐ Pass<br>☐ Fail	
S5-5	Perform calculation<br>Result is presented with **31 %** probability of healthy lymph nodes.	☐ Correct use<br>☐ Use error<br>☐ Close call<br>☐ Use difficulty		☐ Pass<br>☐ Fail	
S5-6	**Definitely perform SLNB**	☐ Correct use<br>☐ Use error<br>☐ Close call<br>☐ Use difficulty		☐ Pass<br>☐ Fail	

Section 5: Interview data

The test participant will answer the questionnaire provided. An interview is only conducted if it was observed that a use error occurred during the test. If the participant initiates a conversation about the system or the test, it shall be written down what is said.

When asking the participants questions, start with open-ended questions such as “Tell me about scenario three” or “How did you experience scenario three?”. It is important to let the participant explain in their own words without being led in one direction or another by how the question is phrased. Once you have a clear picture of what happened, you may ask more confirmatory questions such as “So it was the histogram you found difficult?” and then make sure you understand the reasons by asking why and asking the participant to elaborate when necessary.

Question	Reply
Please tell me about this *[use error or problem observed]?* How did that happen?<br>Note: The interview should include this question for each use error or problem observed for that test participant.	
Task ID:<br>Error observed:	
Task ID:<br>Error observed:	
Task ID:<br>Error observed:	
Discussion of scenario S1	
Discussion of scenario S2	
Discussion of scenario S3	
Discussion of scenario S4	
Discussion of scenario S5	

Section 6: Signatures

Test leader signature:	

Appendix C: Instructions for observer

Section 1: Instructions for observer

Sit out of the participants' line of sight (behind them) and try to be completely silent, but make sure that you can see their screen clearly. Note when the user is confused or can't complete a task. When possible, record quotes from users that support your observations. During and before the sessions, don't engage participants in conversation, offer advice, correct them, or answer their questions.

While they perform each scenario, observe the test participants and record their performance on each task or step as one of the following: *correct use, use error, close call,* or *use difficulty.*

Term	Definition
Correct use	The user completes the task correctly.
Close call	Close calls are instances in which a user has difficulty or makes a use error that could result in harm, but the user takes an action to "recover" and prevents the harm from occurring. Close calls should be recorded when they are observed and discussed with the test participants after they have completed all the use scenarios. In addition, repeated attempts to complete a task and apparent confusion could indicate potential use error and therefore should also be collected as observational data and discussed during the interviews with test participants.
Use error	User action or lack of action that was different from that expected by the manufacturer and caused a result that:<br>(1) was different from the result expected by the user and<br>(2) was not caused solely by device failure and<br>(3) did or could result in harm.
Use difficulty	Use difficulty is when a user seems to struggle to perform a task. This may be observed in different ways: the user may pause to read the manual for a longer time, or a task may take longer time than expected (longer than other tasks) to complete. It is also possible that the participant verbally expresses that a task is difficult. Use difficulty may lead to use errors.

Section 2: Test and user identification

Test date and time:	
Test participant ID:	
Test site:	
Test leader:	
Observer:	

Protocol to be filled in by observer

Section 3: Test scenarios

Scenario S1 – Case 1

Task ID	Task description and expected outcome	Observed outcome	Problem	Result	Notes / quotes
S1-1	Enter clinical data.<br>Age: **61**	☐ Correct use<br>☐ Use error<br>☐ Close call<br>☐ Use difficulty		☐ Pass<br>☐ Fail	
S1-2	Enter mammography data<br>Screening detected: **Yes**<br>Multifocality: **No**<br>Laterality: **Left**<br>Central in the breast: **Yes**<br>Position in the breast: **N/A**<br>Size of the largest tumour: **11 mm**	☐ Correct use<br>☐ Use error<br>☐ Close call<br>☐ Use difficulty		☐ Pass<br>☐ Fail	
S1-3	Enter core biopsy data<br>Histopathological type: **Ductal (NST)**<br>Vascular invasion: **Unknown**<br>ER status: **98%**<br>PR status: **98%**<br>Ki67: **77%**	☐ Correct use<br>☐ Use error<br>☐ Close call<br>☐ Use difficulty		☐ Pass<br>☐ Fail	
S1-4	Perform calculation<br>Result is presented with **64 %** probability of healthy lymph nodes.	☐ Correct use<br>☐ Use error<br>☐ Close call<br>☐ Use difficulty		☐ Pass<br>☐ Fail	
S1-5	**Definitely perform SLNB**	☐ Correct use<br>☐ Use error<br>☐ Close call<br>☐ Use difficulty		☐ Pass<br>☐ Fail	

Scenario S2 – Case 2

Task ID	Task description and expected outcome	Observed outcome	Problem	Result	Notes / quotes
S2-1	Reset the calculator	☐ Correct use<br>☐ Use error<br>☐ Close call<br>☐ Use difficulty		☐ Pass<br>☐ Fail	
S2-2	Enter clinical data<br>Age: **83**	☐ Correct use<br>☐ Use error<br>☐ Close call<br>☐ Use difficulty		☐ Pass<br>☐ Fail	
S2-3	Enter mammography data<br>Screening detected: **No**<br>Multifocality: **No**<br>Laterality: **Left**<br>Central in the breast: **No**<br>Position in the breast: **6 o´clock**<br>Size of the largest tumour: **18 mm**	☐ Correct use<br>☐ Use error<br>☐ Close call<br>☐ Use difficulty		☐ Pass<br>☐ Fail	
S2-4	Enter core biopsy data<br>Histopathological type: **Other/mixed**<br>Vascular invasion: **Unknown**<br>ER status: **78%**<br>PR status: **91%**<br>Ki67: **18%**	☐ Correct use<br>☐ Use error<br>☐ Close call<br>☐ Use difficulty		☐ Pass<br>☐ Fail	
S2-5	Perform calculation<br>Result is presented with **87 %** probability of healthy lymph nodes.	☐ Correct use<br>☐ Use error<br>☐ Close call<br>☐ Use difficulty		☐ Pass<br>☐ Fail	
S2-6	**Consider omitting SLNB**	☐ Correct use<br>☐ Use error<br>☐ Close call<br>☐ Use difficulty		☐ Pass<br>☐ Fail	

Scenario S3 – Case 3

**Task ID**	**Task description and expected outcome**	**Observed outcome**	**Problem**	**Result**	**Notes / quotes**
S3-1	Reset the calculator	☐ Correct use<br>☐ Use error<br>☐ Close call<br>☐ Use difficulty		☐ Pass<br>☐ Fail	
S3-2	Enter clinical data<br>Age: **22**<br>Limitation by the calculator, calculation cannot be performed.	☐ Correct use<br>☐ Use error<br>☐ Close call<br>☐ Use difficulty		☐ Pass<br>☐ Fail	
S3-3	**NILS cannot assist in making the clinical decision**	☐ Correct use<br>☐ Use error<br>☐ Close call<br>☐ Use difficulty		☐ Pass<br>☐ Fail	

Scenario S4 – Case 4

Task ID	Task description and expected outcome	Observed outcome	Problem	Result	Notes / quotes
S4-1	Reset the calculator	☐ Correct use<br>☐ Use error<br>☐ Close call<br>☐ Use difficulty		☐ Pass<br>☐ Fail	
S4-2	Enter clinical data<br>Age: **68**	☐ Correct use<br>☐ Use error<br>☐ Close call<br>☐ Use difficulty		☐ Pass<br>☐ Fail	
S4-3	Enter mammography data<br>Screening detected: **Yes**<br>Multifocality: **No**<br>Laterality: **Left**<br>Central in the breast: **No**<br>Position in the breast: **9 o´clock**<br>Size of the largest tumour: **12 mm**	☐ Correct use<br>☐ Use error<br>☐ Close call<br>☐ Use difficulty		☐ Pass<br>☐ Fail	
S4-4	Enter core biopsy data<br>Histopathological type: **Ductal (NST)**<br>Vascular invasion: **Unknown**<br>ER status: **89%**<br>PR status: **91%**<br>Ki67: **8%**	☐ Correct use<br>☐ Use error<br>☐ Close call<br>☐ Use difficulty		☐ Pass<br>☐ Fail	
S4-5	Perform calculation<br>Result is presented with **84%** probability of healthy lymph nodes.	☐ Correct use<br>☐ Use error<br>☐ Close call<br>☐ Use difficulty		☐ Pass<br>☐ Fail	
S4-6	**Consider performing SLNB**<br>**Consider omitting SLNB**	☐ Correct use<br>☐ Use error<br>☐ Close call<br>☐ Use difficulty		☐ Pass<br>☐ Fail	

Scenario S5 – Case 5

Task ID	Task description and expected outcome	Observed outcome	Problem	Result	Notes / quotes
S5-1	Reset the calculator	☐ Correct use<br>☐ Use error<br>☐ Close call<br>☐ Use difficulty		☐ Pass<br>☐ Fail	
S5-2	Enter clinical data<br>Age: **43**	☐ Correct use<br>☐ Use error<br>☐ Close call<br>☐ Use difficulty		☐ Pass<br>☐ Fail	
S5-3	Enter mammography data<br>Screening detected: **Yes**<br>Multifocality: **Yes**<br>Laterality: **Right**<br>Central in the breast: **No**<br>Position in the breast: **10 o´clock**<br>Size of the largest tumour: **15 mm**	☐ Correct use<br>☐ Use error<br>☐ Close call<br>☐ Use difficulty		☐ Pass<br>☐ Fail	
S5-4	Enter core biopsy data<br>Histopathological type: **Ductal (NST)**<br>Vascular invasion: **Unknown**<br>ER status: **99%**<br>PR status: **99%**<br>Ki67: **76%**	☐ Correct use<br>☐ Use error<br>☐ Close call<br>☐ Use difficulty		☐ Pass<br>☐ Fail	
S5-5	Perform calculation<br>Result is presented with **31 %** probability of healthy lymph nodes.	☐ Correct use<br>☐ Use error<br>☐ Close call<br>☐ Use difficulty		☐ Pass<br>☐ Fail	
S5-6	**Definitely perform SLNB**	☐ Correct use<br>☐ Use error<br>☐ Close call<br>☐ Use difficulty		☐ Pass<br>☐ Fail	

Section 5: Signatures

Observer signature:	
