# Supplementary material for: Human factors validation study of an artificial neural network‑based preoperative decision‑support tool for noninvasive lymph node staging (NILS) in women with primary breast cancer (ISRCTN99301435)
Source: BMC Cancer. 2026 May 28;26:691. doi: 10.1186/s12885-026-16161-5 (PMC13221748; doi:10.1186/s12885-026-16161-5)
Supplement: Supplementary file 2 — Supplementary Material 2. Definitions applied during the usability testing for observed outcomes for each predefined task. [file 12885_2026_16161_MOESM2_ESM.docx]

Supplement 2. Definitions applied during the usability testing for observed outcomes for each predefined task

| **Term** | **Definition** |
| --- | --- |
| Correct use | The user completes the task correctly. |
| Close call | Close calls are instances in which a user has difficulty or makes a use error that could result in harm, but the user takes an action to “recover” and prevents the harm from occurring. Close calls should be recorded when they are observed and discussed with the test participants after they have completed all the use scenarios. In addition, repeated attempts to complete a task and apparent confusion could indicate potential use error and therefore should also be collected as observational data and discussed during the interviews with test participants. |
| Use error | User action or lack of action that was different from that expected by the manufacturer and caused a result that:  (1) was different from the result expected by the user and  (2) was not caused solely by device failure and  (3) did or could result in harm. |
| Use difficulty | Use difficulty is when a user seems to struggle to perform a task. This may be observed in different ways: the user may pause to read the manual for a longer time, or a task may take longer time than expected (longer than other tasks) to complete. It is also possible that the participant verbally expresses that a task is difficult. Use difficulty may lead to use errors. |
