# Supplementary material for: Human factors validation study of an artificial neural network‑based preoperative decision‑support tool for noninvasive lymph node staging (NILS) in women with primary breast cancer (ISRCTN99301435)
Source: BMC Cancer. 2026 May 28;26:691. doi: 10.1186/s12885-026-16161-5 (PMC13221748; doi:10.1186/s12885-026-16161-5)
Supplement: Supplementary file 3 — Supplementary Material 3. Specifications of potential use errors that can be avoided through safe devise design. [file 12885_2026_16161_MOESM3_ESM.docx]

**Supplement 3. Specifications of potential use errors that can be avoided through safe devise design**

| **Hazard** | **Hazardous situation** | **Cause** |
| --- | --- | --- |
| Complex user interface | False estimated indication of benign SLN* | User interface is complex and allows misinterpretation |
|  | False estimated indication of benign SLN | User is confused when the user interface reports the probability of benign sentinel lymph node status instead of risk/probability for metastatic SLN status. This refer to the probability of "no-disease" which is not commonly used in risk prediction models. Because of this the probability and threshold can be misinterpreted, above threshold is in fact representing "no-disease" state. |
|  | False estimated indication of metastatic SLN | The interpreting physician does not understand the graph background data (histogram) and do not know how to interpret the results. |
|  | False estimated indication of metastatic SLN | Colours are used in a way that is not conventional and intuitive. The physician is confused how to interpret the results if signal colours are used inconsistently. |
|  | False estimated indication of metastatic SLN | Physician is unsure how to interpret data that is very close to the threshold or is unsure if the results can be used in order to omit SLNB |
| Use error | False estimated indication of benign SLN | The physician using NILS selects "Other/Mixed" as type in histopathological subtype with the intention of selecting "Unknown" for imputation. |
| Information provided by the manufacturer | False estimated indication of benign SLN | Incomplete instructions for use |
|  | False estimated indication of benign SLN | Inadequate description of performance characteristics |
|  | False estimated indication of benign SLN | Inadequate specification of intended use |
|  | False estimated indication of benign SLN | Inadequate disclosure of limitations |
|  | False estimated indication of benign SLN | Information provided by the manufacturer is not readily available and therefore omitted/missed at time point of NILS calculator |

* False estimated indication of benign sentinel lymph node is the most important harm to avoid.

The risk management procedure is based on the guidance provided in ISO 14971 International Electrotechnical Commission. Medical Devices - Part 2: Guidance on the Application of Usability Engineering to Medical Devices. ed 1.0, IEC, Tr; 2016:62366-2:2016. https://www.iso.org/standard/69126.html.
