# Supplementary material for: Human factors validation study of an artificial neural network‑based preoperative decision‑support tool for noninvasive lymph node staging (NILS) in women with primary breast cancer (ISRCTN99301435)
Source: BMC Cancer. 2026 May 28;26:691. doi: 10.1186/s12885-026-16161-5 (PMC13221748; doi:10.1186/s12885-026-16161-5)
Supplement: Supplementary file 4 — Supplementary Material 4. Frequency of observed outcome and result, analysis per task description. [file 12885_2026_16161_MOESM4_ESM.docx]

**Supplement 4. Frequency of observed outcome and result, analysis per task description**

|  | Task description | Potential harm | Observed outcome, N (%) | | | | Result, N (%) | |
| --- | --- | --- | --- | --- | --- | --- | --- | --- |
|  |  |  | Correct use | Use error | Use difficulty | Close call | Pass | Fail |
| 1. | Reset the calculator | False estimated indication of benign SLN | 68 (85) | 12 (15) | 0 (0) | 0 (0) | 68 (85) | 12 (15) |
| 2. | Enter clinical data | False estimated indication of benign SLN | 83 (83) | 1 (1) | 16 (16) | 0 (0) | 99 (99) | 1 (1) |
| 3. | Enter mammography data | False estimated indication of benign SLN | 77 (96) | 1 (1) | 2 (3) | 0 (0) | 79 (99) | 1 (1) |
| 4. | Enter core biopsy data | False estimated indication of benign SLN | 73 (91) | 7 (9) | 0 (0) | 0 (0) | 73 (91) | 7 (9) |
| 5. | Perform calculation | - | 71 (89) | 9 (11) | 0 (0) | 0 (0) | 71 (89*) | 9 (11) |
| 6. | Fill out the questionnaire with the appropriate clinical pathway selected considering the results of NILS in combination with all other available information of the case. | False estimated indication of benign SLN | 94 (94) | 4 (4) | 1 (1) | 1 (1) | 96 (96) | 4 (4) |

Cell color indications: Green indicates a task result categorized as "pass" (meeting or exceeding the pre-defined acceptance criteria of ≥ 90%); Red indicates a task result categorized as "fail" (falling below the pre-defined acceptance criteria of ≥ 90%).

*The “Perform calculation” were below the acceptance criteria of ≥ 90% for the first of the four cases when analyzed per task. However, the summary of all cases resulted in a percentage just below the threshold. Altogether, reflecting a learning curve.
