# Supplementary material for: Human factors validation study of an artificial neural network‑based preoperative decision‑support tool for noninvasive lymph node staging (NILS) in women with primary breast cancer (ISRCTN99301435)
Source: BMC Cancer. 2026 May 28;26:691. doi: 10.1186/s12885-026-16161-5 (PMC13221748; doi:10.1186/s12885-026-16161-5)
Supplement: Supplementary file 5 — Supplementary Material 5. The System Usability Scale (SUS) to assess participants’ levels of agreement with the overall usability of the system. Test participants N=20 (results per question). [file 12885_2026_16161_MOESM5_ESM.docx]

**Supplement 5. The System Usability Scale (SUS) to assess participants’ levels of agreement with the overall usability of the system. Test participants N=20 (results per question)**

Please note that odd-numbered items are positive and even-numbered items are negative.

|  |  |  | Likert scale 1-5:  1, “Strongly Disagree”; 5 “Strongly Agree“ | SUS score |
| --- | --- | --- | --- | --- |
|  | | Theme from SUS | Average point  mean (SD)  median (range) | Average score mean  median |
| 1 | I think that I would like to use this system frequently | Readiness to use | 4.15 (0.75)  4.00 (3.00-5.00) | 3.15  3.00 |
| 2 | I found the system unnecessarily complex | Tool simplicity | 1.25 (0.44)  1.00 (1.00-2.00) | 3.75  4.00 |
| 3 | I thought the system was easy to use | Ease of use | 4.55 (0.61)  5.00 (3.00-5.00) | 3.55  4.00 |
| 4 | I think that I would need the support of a technical person to be able to use this system | Need for support to use tool | 1.15 (0.37)  1.00 (1.00-2.00) | 3.85  4.00 |
| 5 | I found the various functions in this system were well integrated | Understanding the input parameters | 4.40 (0.60)  4.00 (3.00-5.00) | 3.40  3.00 |
| 6 | I thought there was too much inconsistency in this system | Clarity of the tool | 1.25 (0.55)  1.00 (1.00-3.00) | 3.75  4.00 |
| 7 | I would imagine that most people would learn to use this system very quickly | Need for technical details | 4.70 (0.47)  5.00 (4.00-5.00) | 3.70  4.00 |
| 8 | I found the system very awkward to use | Easier-to-use tool | 1.20 (0.41)  1.00 (1.00-2.00) | 3.80  4.00 |
| 9 | I felt very confident using the system | Usability confidence | 4.05 (0.61)  4.00 (3.00-5.00) | 3.05  3.00 |
| 10 | I needed to learn a lot of things before I could get going with this system | Easy to use for everyone | 1.20 (0.70)  1.00 (1.00-4.00) | 3.80  4.00 |
|  |  |  |  |  |
|  | SUS score, mean | (3.15+3.75+3.55+3.85+3.40+3.75+3.70+3.80+3.05+3.80) x 2.5  Σ score: 89.5* | | |
|  | SUS score, median | (3+4+4+4+3+4+4+4+3+4) x 2.5  Σ score: 92.5 | | |

*Categorized as “Excellent”, according to the commonly used Adjective Rating Scale (A. Bangor et al, 2009). The Adjective Rating Scale: “Best imaginable” ≥ 90.9; “Excellent” ≥ 85.5; “Good” ≥ 71.4; “OK/Fair” ≥ 50.9; “Poor” ≥ 35.7; “Awful” ≥ 20.3; and “Worst imaginable” ≥ 12.5.
