# Supplementary material for: Human factors validation study of an artificial neural network‑based preoperative decision‑support tool for noninvasive lymph node staging (NILS) in women with primary breast cancer (ISRCTN99301435)
Source: BMC Cancer. 2026 May 28;26:691. doi: 10.1186/s12885-026-16161-5 (PMC13221748; doi:10.1186/s12885-026-16161-5)
Supplement: Supplementary file 6 — Supplementary Material 6. The System Usability Scale (SUS) to assess participants’ level of agreement with the overall usability of the system (results per test participant). [file 12885_2026_16161_MOESM6_ESM.docx]

**Supplement 6. The System Usability Scale (SUS) to assess participants’ level of agreement with the overall usability of the system (results per test participant)**

1. The Table shows individual SUS score for each test participant
2. Box Plot, mean SUS score per test participant = 89.4

| Test participant | Question | | | | | | | | | | SUS score |
| --- | --- | --- | --- | --- | --- | --- | --- | --- | --- | --- | --- |
|  | **1** | **2** | **3** | **4** | **5** | **6** | **7** | **8** | **9** | **10** | **Σ** |
| 1 | 2 | 4 | 3 | 4 | 3 | 4 | 4 | 3 | 3 | 4 | 85.0 |
| 2 | 3 | 4 | 2 | 3 | 3 | 3 | 3 | 3 | 3 | 3 | 75.0 |
| 3 | 3 | 4 | 4 | 4 | 3 | 4 | 4 | 4 | 3 | 4 | 92.5 |
| 4 | 3 | 4 | 4 | 4 | 4 | 4 | 4 | 4 | 3 | 4 | 95.0 |
| 5 | 4 | 4 | 4 | 4 | 4 | 4 | 4 | 4 | 3 | 4 | 97.5 |
| 6 | 4 | 4 | 3 | 4 | 4 | 4 | 4 | 3 | 4 | 4 | 95.0 |
| 7 | 3 | 4 | 3 | 4 | 2 | 3 | 3 | 3 | 2 | 1 | 70.0 |
| 8 | 3 | 4 | 4 | 4 | 4 | 4 | 4 | 4 | 3 | 4 | 95.0 |
| 9 | 4 | 4 | 4 | 4 | 4 | 4 | 4 | 4 | 4 | 4 | 100.0 |
| 10 | 3 | 3 | 3 | 4 | 3 | 4 | 3 | 4 | 2 | 4 | 82.5 |
| 11 | 3 | 3 | 4 | 4 | 4 | 2 | 3 | 4 | 3 | 4 | 85.0 |
| 12 | 2 | 4 | 4 | 4 | 3 | 3 | 4 | 4 | 4 | 4 | 90.0 |
| 13 | 2 | 4 | 4 | 4 | 4 | 4 | 3 | 4 | 3 | 4 | 90.0 |
| 14 | 4 | 3 | 4 | 3 | 3 | 4 | 4 | 4 | 3 | 4 | 90.0 |
| 15 | 2 | 4 | 4 | 4 | 4 | 4 | 4 | 4 | 2 | 4 | 90.0 |
| 16 | 4 | 3 | 4 | 3 | 3 | 4 | 4 | 4 | 3 | 4 | 90.0 |
| 17 | 4 | 4 | 3 | 4 | 3 | 4 | 4 | 4 | 3 | 3 | 90.0 |
| 18 | 4 | 4 | 4 | 4 | 4 | 4 | 4 | 4 | 4 | 4 | 100.0 |
| 19 | 3 | 3 | 3 | 4 | 3 | 4 | 4 | 4 | 3 | 4 | 87.5 |
| 20 | 3 | 4 | 3 | 4 | 3 | 4 | 3 | 4 | 3 | 4 | 87.5 |
| SUS score per test participants mean |  |  |  |  |  |  |  |  |  |  | Σ score: 89.4* |
| SUS score per test participant median |  |  |  |  |  |  |  |  |  |  | Σ score: 90.0 |

*Categorized as “Excellent”, according to the commonly used Adjective Rating Scale (A. Bangor et al, 2009). The Adjective Rating Scale: “Best imaginable” ≥ 90.9; “Excellent” ≥ 85.5; “Good” ≥ 71.4; “OK/Fair” ≥ 50.9; “Poor” ≥ 35.7; “Awful” ≥ 20.3; and “Worst imaginable” ≥ 12.5.


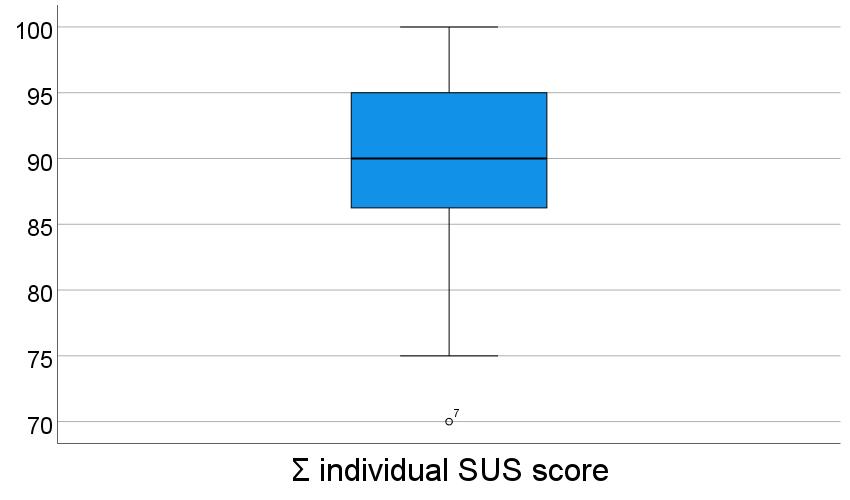


b)
