# Supplementary material for: Human factors validation study of an artificial neural network‑based preoperative decision‑support tool for noninvasive lymph node staging (NILS) in women with primary breast cancer (ISRCTN99301435)
Source: BMC Cancer. 2026 May 28;26:691. doi: 10.1186/s12885-026-16161-5 (PMC13221748; doi:10.1186/s12885-026-16161-5)
Supplement: Supplementary file 7 — Supplementary Material 7. After-Scenario Questionnaire (ASQ) to assess the participants’ level of satisfaction with the NILS model. Test participants N=20 (results per question). [file 12885_2026_16161_MOESM7_ESM.docx]

**Supplement 7. After-Scenario Questionnaire (ASQ) to assess the participants’ level of satisfaction with the NILS model. Test participants N=20 (results per question).**

|  |  | Likert scale 1-7:  1, “Strongly Disagree”; 7, “Strongly Agree“ |
| --- | --- | --- |
|  | | Average point  mean (SD)  median (range) |
| 1 | Overall, I am satisfied with the ease of completing the tasks in this scenario. | 6.20 (0.70)  6.00 (5.00-7.00) |
| 2 | Overall, I am satisfied with the amount of time it took to complete the tasks in this scenario. | 6.45 (0.61)  6.50 (5.00-7.00) |
| 3 | Overall, I am satisfied with the support information (on-line help, documentation) when completing the tasks. | 6.25 (0.97)  6.50 (4.00-7.00) |
|  | Total | mean: 6.30  median: 6.50 |
