# Supplementary material for: Human factors validation study of an artificial neural network‑based preoperative decision‑support tool for noninvasive lymph node staging (NILS) in women with primary breast cancer (ISRCTN99301435)
Source: BMC Cancer. 2026 May 28;26:691. doi: 10.1186/s12885-026-16161-5 (PMC13221748; doi:10.1186/s12885-026-16161-5)
Supplement: Supplementary file 8 — Supplementary Material 8. Implemented alterations of the interface of the NILS model and results of repeated usability testing. Test participants N=6. [file 12885_2026_16161_MOESM8_ESM.docx]

**Supplement 8. a) Implemented alterations of the interface of the NILS model b) Results of repeated usability testing**

**a)**

| **#** | **Issue** | **Solution** |
| --- | --- | --- |
| 1 | When the age is outside the accepted range the cell becomes yellow, however, still possible to enter a value. If notice is not taken, unnecessary work is done inserting all other variables. It will, however, not be possible to make a NILS calculation. | The cell is highlighted in red, and an explanatory error message is shown underneath the field to the user. A screenshot of the updated interface is shown below.  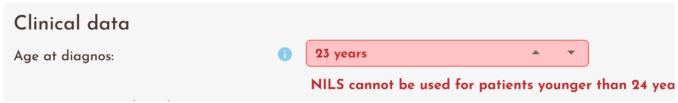 |
| 2 | If notice is not taken that tumor size is out of range, unnecessary work is done inserting all other variables. It will, however, not be possible to make a NILS calculation. | The cell is highlighted in red, and an explanatory error message is shown underneath the field to the user. A screenshot of the updated interface is shown below.  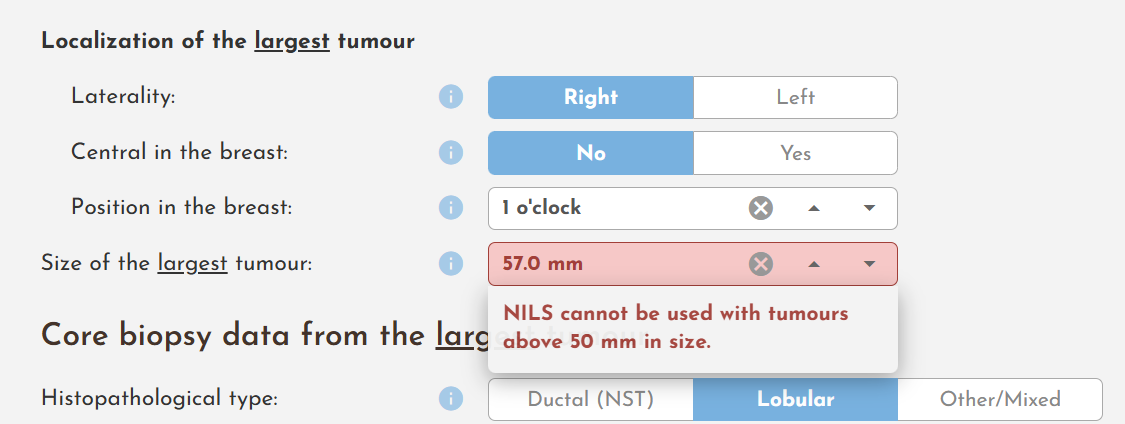 |
| 3 | The reset button is placed at the bottom of the calculator. When making the calculation the user get directed automatically to the histogram, which is displayed in the upper part of the calculator and the reset button then falls out of sight. | The reset button is moved to the top of the interface and highlighted using more prominent colors (blue and white), together with the addition of a reset icon. A screenshot of the updated interface is shown below.  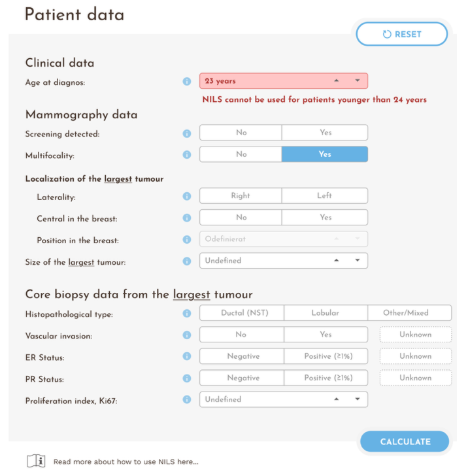 |
| 4 | The scrolling can alter the inserted value, and if not identified and corrected cause the calculated value to be wrong. | The cell component was redesigned so that scrolling no longer alters entered values. |
| 5 | When struggling with the decision on which tumor variables to enter in case of multifocality and how a central tumor is defined, the information button was seldom identified. | The information button was repositioned to enhance visibility and highlighted using more prominent colors (blue and white). A clearer link to the Instructions for Use was added. When multifocality is selected, the heading updates dynamically to indicate that the entered information refers to the largest tumor. A screenshot of the updated interface is shown below.  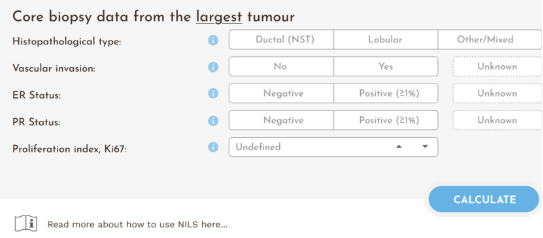 |
| 6 | The cut-off value can be misinterpreted as the calculated value if the y-axis and the text below only are viewed. | The cut‑off value was added to the histogram, and the legend was updated to emphasize the resulting percentages rather than the cut‑off value. A screenshot of the updated interface is shown below.      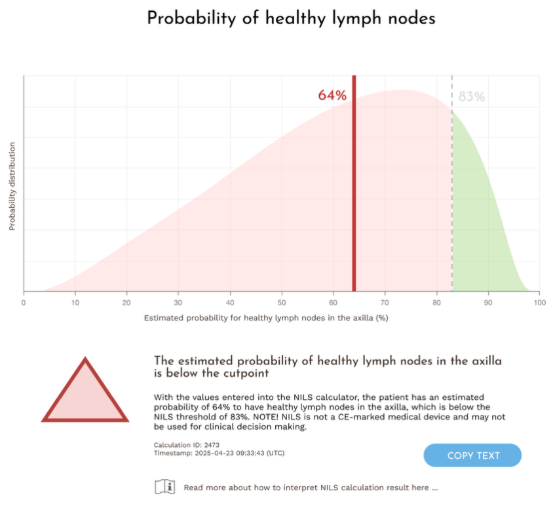 |
| 7 | For correct interpretation of the presented histogram/result, proper labelling is warranted. | A comprehensive Instruction for Use with clear guidance, information on result interpretation, and clarification of common misconceptions was developed. A screenshot of the updated interface is shown below.  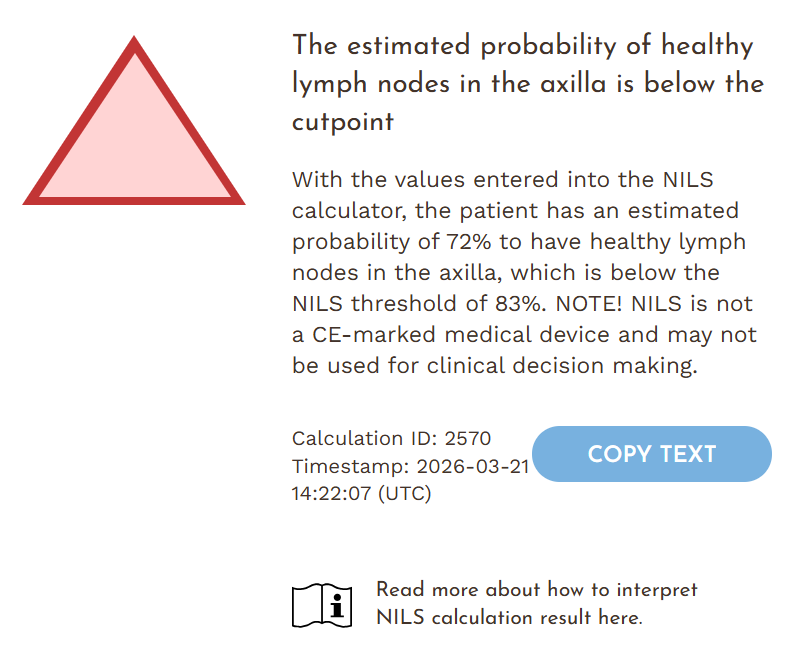 |
| 8 | If disclaimers are not noted, the NILS model can be used by mistake on the wrong patient population. | A clearer link to the Instruction for Use was added. A screenshot of the updated interface is shown below.  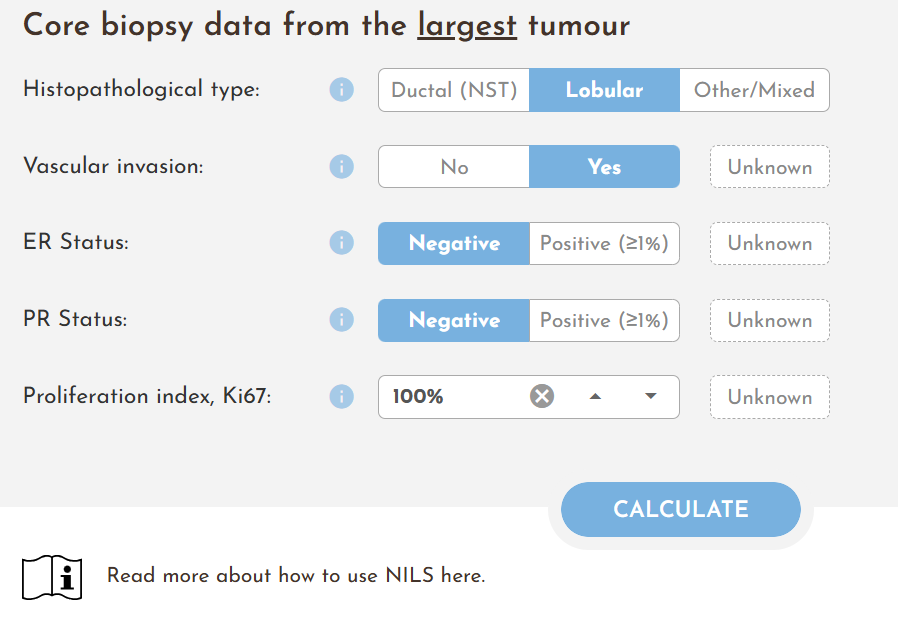 |

**b)**

Following implementation of these changes, a cohort of N=6 test participants evaluated the usability of the updated interface. The demographics and results are presented below.

Supplementary Material 8b.1: Self-reported characteristics of usability testing participants
Supplementary Material 8b.2: Frequency of observed outcome and result, analysis per task  
Supplementary Material 8b.3: Frequency of observed outcome and result, analysis per task description
Supplementary Material 8b.4: The System Usability Scale (SUS) to assess participants’ levels of agreement with the overall usability of the system. Test participants N=6 (results per question
Supplementary Material 8b.5: The System Usability Scale (SUS) to assess participants’ level of agreement with the overall usability of the system (results per test participant)
Supplementary Material 8b.6: After-Scenario Questionnaire (ASQ) to assess the participants’ level of satisfaction with the NILS model. Test participants N=20 (results per question).

**Supplementary Material 8b.1. Self-reported characteristics of usability testing participants**

| Physician characteristics | | **Usability testing participants**  **N=6 (%)** |
| --- | --- | --- |
| Specialty | Surgeon | 4 (67) |
|  | Oncologist | 2 (33) |
| Completed years at specialist (years), median (IQR) |  | 20 (11-25) |
| Use of any tool/system for prediction in clinical work | Yes | 6 (100) |
|  | No | 0 (0) |
| 1. If, yes – which? |  | N = 6, Predict (100) |
| Workplace (Hospital) | University Hospital | 4 (67) |
|  | Regional Hospital 1 | x |
|  | Regional Hospital 2 | x |
|  | Regional Hospital 3 | x |
|  | Regional hospital 4 | 2 (33) |
| Highest academic degree | MD | 1 (17) |
|  | MD + PhD | 3 (50) |
|  | Associate Professor | 2 (33) |
|  | Professor | - |
| Gender | Male | 4 (67) |
|  | Female | 2 (33) |
|  | Other | - |
|  | Do not want to say | - |
| Age (years), median (IQR) |  | 57 (49-63) |

Abbreviations: IQR, interquartile range; MD, Doctor of Medicine; PhD, Doctor of Philosophy

**Supplementary Material 8b.2. Frequency of observed outcome and result, analysis per task**

|  | Description | Observed outcome, N (%) | | | | Result, N (%) | |
| --- | --- | --- | --- | --- | --- | --- | --- |
| Scenario |  | Correct use | Use error | Use difficulty | Close call | Pass | Fail |
| 1 | Reset the calculator* | N/A | N/A | N/A | N/A | N/A | N/A |
|  | Enter clinical data | 6 (100) | 0 (0) | 0 (0) | 0 (0) | 6 (100) | 0 (0) |
|  | Enter mammography data | 6 (100) | 0 (0) | 0 (0) | 0 (0) | 6 (100) | 0 (0) |
|  | Enter core biopsy data | 6 (100) | 0 (0) | 0 (0) | 0 (0) | 6 (100) | 0 (0) |
|  | Perform calculation | 6 (100) | 0 (0) | 0 (0) | 0 (0) | 6 (100) | 0 (0) |
|  | Select the appropriate clinical pathway | 6 (100) | 0 (0) | 0 (0) | 0 (0) | 6 (100) | 0 (0) |
| 2 | Reset the calculator | 6 (100) | 0 (0) | 0 (0) | 0 (0) | 6 (100) | 0 (0) |
|  | Enter clinical data | 6 (100) | 0 (0) | 0 (0) | 0 (0) | 6 (100) | 0 (0) |
|  | Enter mammography data | 6 (100) | 0 (0) | 0 (0) | 0 (0) | 6 (100) | 0 (0) |
|  | Enter core biopsy data | 6 (100) | 0 (0) | 0 (0) | 0 (0) | 6 (100) | 0 (0) |
|  | Perform calculation | 6 (100) | 0 (0) | 0 (0) | 0 (0) | 6 (100) | 0 (0) |
|  | Select the appropriate clinical pathway | 6 (100) | 0 (0) | 0 (0) | 0 (0) | 6 (100) | 0 (0) |
| 3 | Reset the calculator | 6 (100) | 0 (0) | 0 (0) | 0 (0) | 6 (100) | 0 (0) |
|  | Enter clinical data | 4 (67) | 0 (0) | 2 (33) | 0 (0) | 6 (100) | 0 (0) |
|  | Select the appropriate clinical pathway | 6 (100) | 0 (0) | 0 (0) | 0 (0) | 6 (100) | 0 (0) |
| 4 | Reset the calculator | 6 (100) | 0 (0) | 0 (0) | 0 (0) | 6 (100) | 0 (0) |
|  | Enter clinical data | 6 (100) | 0 (0) | 0 (0) | 0 (0) | 6 (100) | 0 (0) |
|  | Enter mammography data | 6 (100) | 0 (0) | 0 (0) | 0 (0) | 6 (100) | 0 (0) |
|  | Enter core biopsy data | 6 (100) | 0 (0) | 0 (0) | 0 (0) | 6 (100) | 0 (0) |
|  | Perform calculation | 6 (100) | 0 (0) | 0 (0) | 0 (0) | 6 (100) | 0 (0) |
|  | Select the appropriate clinical pathway | 6 (100) | 0 (0) | 0 (0) | 0 (0) | 6 (100) | 0 (0) |
| 5 | Reset the calculator | 6 (100) | 0 (0) | 0 (0) | 0 (0) | 6 (100) | 0 (0) |
|  | Enter clinical data | 6 (100) | 0 (0) | 0 (0) | 0 (0) | 6 (100) | 0 (0) |
|  | Enter mammography data | 6 (100) | 0 (0) | 0 (0) | 0 (0) | 6 (100) | 0 (0) |
|  | Enter core biopsy data | 5 (83) | 1 (17) | 0 (0) | 0 (0) | 5 (83) | 1 (17) |
|  | Perform calculation | 5 (83) | 1 (17) | 0 (0) | 0 (0) | 5 (83) | 1 (17) |
|  | Select the appropriate clinical pathway | 6 (100) | 0 (0) | 0 (0) | 0 (0) | 6 (100) | 0 (0) |

 
*N/A: Resetting the calculator was not applicable in scenario 1.

**Supplementary Material 8b.3. Frequency of observed outcome and result, analysis per task description**

|  | **Task description** | **Potential harm** | **Observed outcome, N (%)** | | | | **Result, N (%)** | |
| --- | --- | --- | --- | --- | --- | --- | --- | --- |
|  |  |  | Correct use | Use error | Use difficulty | Close call | Pass | Fail |
| **1.** | Reset the calculator | False estimated indication of benign SLN | 24 (100) | 0 (0) | 0 (0) | 0 (0) | 24 (100) | 0 (0) |
| **2.** | Enter clinical data | False estimated indication of benign SLN | 30 (100) | 0 (0) | 0 (0) | 0 (0) | 30 (100) | 0 (0) |
| **3.** | Enter mammography data | False estimated indication of benign SLN | 24 (100) | 0 (0) | 0 (0) | 0 (0) | 24 (100) | 0 (0) |
| **4.** | Enter core biopsy data | False estimated indication of benign SLN | 23 (96) | 1 (4) | 0 (0) | 0 (0) | 23 (96) | 1 (4) |
| **5.** | Perform calculation | - | 23 (96) | 1 (4) | 0 (0) | 0 (0) | 23 (96) | 1 (4) |
| **6.** | Fill out the questionnaire with the appropriate clinical pathway selected considering the results of NILS in combination with all other available information of the case. | False estimated indication of benign SLN | 30 (100) | 0 (0) | 0 (0) | 0 (0) | 30 (100) | 0 (0) |

**Supplementary Material 8b.4. The System Usability Scale (SUS) to assess participants’ levels of agreement with the overall usability of the system. Test participants N=6 (results per question)**

Please note that odd-numbered items are positive and even-numbered items are negative.

|  |  |  | **Likert scale 1-5:**  **1, “Strongly Disagree”; 5 “Strongly Agree“** | **SUS score** |
| --- | --- | --- | --- | --- |
|  | | Theme from SUS | Average point  mean (SD)  median (range) | Average score  mean  median |
| **1** | I think that I would like to use this system frequently | Readiness to use | 4.67 (0.52)  5.00 (4.00-5.00) | 3.67  4.00 |
| **2** | I found the system unnecessarily complex | Tool simplicity | 1.17 (0.41)  1.00 (1.00-2.00) | 3.83  4.00 |
| **3** | I thought the system was easy to use | Ease of use | 4.83 (0.41)  5.00 (4.00-5.00) | 3.83  4.00 |
| **4** | I think that I would need the support of a technical person to be able to use this system | Need for support to use tool | 1.00 (0.00)  1.00 (0.00) | 4.00  4.00 |
| **5** | I found the various functions in this system were well integrated | Understanding the input parameters | 4.83 (0.41)  4.00 (4.00-5.00) | 3.83  4.00 |
| **6** | I thought there was too much inconsistency in this system | Clarity of the tool | 1.33 (0.82)  1.00 (1.00-3.00) | 3.67  4.00 |
| **7** | I would imagine that most people would learn to use this system very quickly | Need for technical details | 5.00 (0.00)  5.00 (0.00) | 4.00  4.00 |
| **8** | I found the system very awkward to use | Easier-to-use tool | 1.00 (0.00)  1.00 (0.00) | 4.00  4.00 |
| **9** | I felt very confident using the system | Usability confidence | 3.83. (1.47)  4.00 (1.00-5.00) | 2.83  3.00 |
| **10** | I needed to learn a lot of things before I could get going with this system | Easy to use for everyone | 1.83 (0.75)  2.00 (1.00-3.00) | 3.17  3.00 |
|  |  |  |  |  |
|  | SUS score, mean | (3.67+3.83+3.83+4.00+3.83+3.67+4.00+4.00+2.83+3.17) x 2.5  Σ score: **92.1*** | | |
|  | SUS score, median | (4+4+4+4+4+4+4+4+3+3) x 2.5  Σ score: **95.0** | | |

*Categorized as “Best imaginable”, according to the commonly used Adjective Rating Scale (A. Bangor et al, 2009). The Adjective Rating Scale: “Best imaginable” ≥ 90.9; “Excellent” ≥ 85.5; “Good” ≥ 71.4; “OK/Fair” ≥ 50.9; “Poor” ≥ 35.7; “Awful” ≥ 20.3; and “Worst imaginable” ≥ 12.5.

**Supplementary Material 8b.5. The System Usability Scale (SUS) to assess participants’ level of agreement with the overall usability of the system (results per test participant)**

The Table shows individual SUS score for each test participant

| **Test participant** | **Question** | | | | | | | | | | **SUS score** |
| --- | --- | --- | --- | --- | --- | --- | --- | --- | --- | --- | --- |
|  | **1** | **2** | **3** | **4** | **5** | **6** | **7** | **8** | **9** | **10** | **Σ** |
| **1** | 4 | 4 | 4 | 4 | 4 | 4 | 4 | 4 | 4 | 4 | 100.0 |
| **2** | 4 | 3 | 3 | 4 | 4 | 4 | 4 | 4 | 3 | 3 | 90.0 |
| **3** | 3 | 4 | 4 | 4 | 3 | 4 | 4 | 4 | 3 | 3 | 90.0 |
| **4** | 3 | 4 | 4 | 4 | 4 | 2 | 4 | 4 | 0 | 2 | 77.5 |
| **5** | 4 | 4 | 4 | 4 | 4 | 4 | 4 | 4 | 3 | 3 | 95.0 |
| **6** | 4 | 4 | 4 | 4 | 4 | 4 | 4 | 4 | 4 | 4 | 100 |
| **SUS score per test participants mean** |  |  |  |  |  |  |  |  |  |  | Σ score: **92.1*** |
| **SUS score per test participant median** |  |  |  |  |  |  |  |  |  |  | Σ score: **92.5** |

*Categorized as “Best imaginable”, according to the commonly used Adjective Rating Scale (A. Bangor et al, 2009). The Adjective Rating Scale: “Best imaginable” ≥ 90.9; “Excellent” ≥ 85.5; “Good” ≥ 71.4; “OK/Fair” ≥ 50.9; “Poor” ≥ 35.7; “Awful” ≥ 20.3; and “Worst imaginable” ≥ 12.5.

**Supplementary Material 8b.6. After-Scenario Questionnaire (ASQ) to assess the participants’ level of satisfaction with the NILS model. Test participants N=6 (results per question)**

|  |  | **Likert scale 1-7:**  **1, “Strongly Disagree”; 7, “Strongly Agree“** |
| --- | --- | --- |
|  | | Average point  mean (SD)  median (range) |
| **1** | Overall, I am satisfied with the ease of completing the tasks in this scenario. | 6.83 (0.41)  7.00 (6.00-7.00) |
| **2** | Overall, I am satisfied with the amount of time it took to complete the tasks in this scenario. | 6.83 (0.41)  7.00 (6.00-7.00) |
| **3** | Overall, I am satisfied with the support information (on-line help, documentation) when completing the tasks. | 5.83 (1.94)  6.50 (2.00-7.00) |
|  | Total | mean:**6.50**  median: **7.00** |
